# Multicellular magnetotactic bacteria are genetically heterogeneous consortia with metabolically differentiated cells

**DOI:** 10.1371/journal.pbio.3002638

**Published:** 2024-07-11

**Authors:** George A. Schaible, Zackary J. Jay, John Cliff, Frederik Schulz, Colin Gauvin, Danielle Goudeau, Rex R. Malmstrom, S. Emil Ruff, Virginia Edgcomb, Roland Hatzenpichler

**Affiliations:** 1 Department of Chemistry and Biochemistry, Montana State University, Bozeman, Montana, United States of America; 2 Center for Biofilm Engineering, Montana State University, Bozeman, Montana, United States of America; 3 Thermal Biology Institute, Montana State University, Bozeman, Montana, United States of America; 4 Environmental Molecular Sciences Laboratory, Pacific Northwest National Laboratory, Richland, Washington, United States of America; 5 Department of Energy Joint Genome Institute, Berkeley, California, United States of America; 6 Ecosystems Center and Bay Paul Center, Marine Biological Laboratory, Woods Hole, Massachusetts, United States of America; 7 Woods Hole Oceanographic Institution, Falmouth, Massachusetts, United States of America; 8 Department of Microbiology and Cell Biology, Montana State University, Bozeman, Montana, United States of America; Fred Hutchinson Cancer Research Center, UNITED STATES

## Abstract

Consortia of multicellular magnetotactic bacteria (MMB) are currently the only known example of bacteria without a unicellular stage in their life cycle. Because of their recalcitrance to cultivation, most previous studies of MMB have been limited to microscopic observations. To study the biology of these unique organisms in more detail, we use multiple culture-independent approaches to analyze the genomics and physiology of MMB consortia at single-cell resolution. We separately sequenced the metagenomes of 22 individual MMB consortia, representing 8 new species, and quantified the genetic diversity within each MMB consortium. This revealed that, counter to conventional views, cells within MMB consortia are not clonal. Single consortia metagenomes were then used to reconstruct the species-specific metabolic potential and infer the physiological capabilities of MMB. To validate genomic predictions, we performed stable isotope probing (SIP) experiments and interrogated MMB consortia using fluorescence in situ hybridization (FISH) combined with nanoscale secondary ion mass spectrometry (NanoSIMS). By coupling FISH with bioorthogonal noncanonical amino acid tagging (BONCAT), we explored their in situ activity as well as variation of protein synthesis within cells. We demonstrate that MMB consortia are mixotrophic sulfate reducers and that they exhibit metabolic differentiation between individual cells, suggesting that MMB consortia are more complex than previously thought. These findings expand our understanding of MMB diversity, ecology, genomics, and physiology, as well as offer insights into the mechanisms underpinning the multicellular nature of their unique lifestyle.

## Abstract 2

Multicellular magnetotactic bacteria (MMB) are unique in having no unicellular stage in their life cycle. This study characterizes the genomics and physiology of MMB consortia at single cell resolution, revealing that individual cells are not genetically identical and exhibit significant metabolic differentiation, highlighting their level of complexity.

## Introduction

Multicellular lifeforms are defined as organisms that are built from several or many cells of the same species [1,2]. Beyond this, other characteristics of multicellularity include a specific shape and organization, a lack of individual cell autonomy or competition between cells, and a display of cell-to-cell signaling and coordinated response to external stimuli [3]. The transition from a single cell to a cooperative multicellular organism is an important evolutionary event that has independently occurred at least 50 times across the tree of life [4]. This suggests that the development of multicellularity can occur in any species given proper selective pressure [5,6]. Prior research on the transition of unicellular to multicellular organisms has largely focused on eukaryotic model systems such as choanoflagellates [7], fungi [8], and algae [9]. Multicellularity within the domain bacteria is uncommon as compared to eukaryotes [10], yet this lifestyle likely first evolved approximately >3 billion years ago [11]. Examples of multicellularity within the domain bacteria include filamentous cyanobacteria (e.g., *Anabaena cylindrica*), mycelia-forming actinomyces (e.g., *Streptomyces coelicolor*), swarming myxobacteria (e.g., *Myxococcus xanthus*), centimeter-long cable bacteria (e.g., *Electrothrix* sp.), and the recently discovered liquid-crystal colonies of Neisseriaceae (e.g., *Jeongeupia sacculi* sp. nov. HS-3) [6,12,13]. While capable of multicellular growth, each of these microbes undergoes a unicellular stage at some point in their life cycle.

Currently, the only known example of purportedly obligate multicellularity—an organism without a detectable unicellular stage—within the domain bacteria are several species of multicellular magnetotactic bacteria (MMB; we use the terms “MMB consortia” and “MMB” interchangeably) [14,15]. Historically, MMB have been described as “aggregates” of cells [16], which could imply that individual cells assemble to form a multicellular aggregate, akin to the early stages of biofilm formation [6,16]. In this study, we use the terms “consortium” (singular) and “consortia” (plural) to describe the unique form of multicellularity observed for MMB.

**Fig 1:**
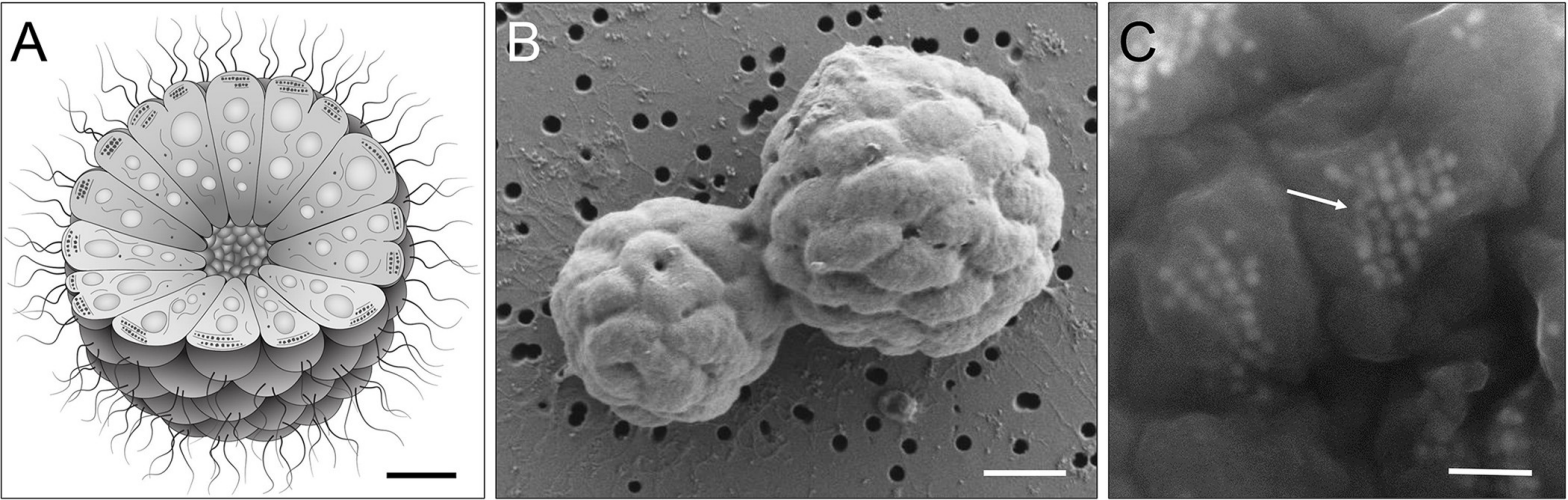
Morphology and structure of MMB. (**A**) Cartoon depicting the morphology and internal organization of an MMB consortium. At the center of each MMB consortium lies an acellular space that is surrounded by a single layer of cells. Each cell harbors magnetosome organelles (black polygons aligned along cytoskeleton-like filaments), compartments for carbon or energy storage (gray circles), as well as other, currently unidentified structures. Scale bar ca. 1 μm. (**B**) SEM image of 2 MMB magnetically enriched from LSSM, possibly undergoing division. Scale bar, 1 μm. (**C**) Backscatter electron microscopy image of magnetosome chains within MMB cells (arrow). Magnetosome minerals appear to have 4–8 visible facets and are approximately 30–60 nm in diameter. Scale bar, 300 nm. Contrast and brightness of image (**C**) was increased for better visualization. LSSM, Little Sippewissett Salt Marsh; MMB, multicellular magnetotactic bacteria; SEM, scanning electron microscopy.

MMB are symmetrical single-species consortia composed of 15 to 86 cells [17] of Desulfobacterota (formerly Deltaproteobacteria) arranged in a single layer enveloping an acellular, central compartment (Fig 1A and 1B). Consortia range in size from 3 to 12 μm in diameter [18–20]. Within the Desulfobacterota, MMB form an uncultured, monophyletic family that is distinct from several physiologically and genetically well-characterized unicellular relatives, suggesting a common ancestor that achieved a multicellular state [21–23].

MMB are unique among bacteria because their life cycle lacks a unicellular stage. Instead, MMB replicate by the entire consortium doubling its cell number and volume before separating into 2 seemingly identical consortia, as has been observed via brightfield, epifluorescence, and scanning electron microscopy (SEM) [15,18,24–26]. Studies using fluorescence in situ hybridization (FISH) have identified MMB exclusively in a multicellular state [14,25,27]. Live-dead staining experiments revealed that when cells become separated from the consortium, for example because of osmotic or mechanical stress, the consortium dismantles. This is followed by an immediate loss of magnetic orientation and motility and eventual loss of membrane integrity, leading to the death of both the separated cells and the consortium [26]. MMB consortia consistently exhibit a high degree of magnetic optimization, excluding the possibility that the consortium is a mere aggregation of cells without underlying self-organization [28,29]. Each cell within the consortium has multiple flagella, resulting in the whole consortium being peritrichously flagellated [19,30]. When environmental conditions change, such as alterations in light exposure or magnetic fields, a coordinated response in motility occurs within fractions of a second [30,31]. This collective response implies inter-cellular communication among individual cells, which is hypothesized to occur through the central acellular volume that the cells surround [18]. Previous work has hypothesized that the absence of a single cell stage in MMB might be necessary to maintain the acellular volume at the center of each MMB or that their larger size is needed to evade predation by protists [15]. Currently, there is no evidence to support or refute these hypotheses.

In addition to their unique obligate multicellular life cycle, MMB have an organelle called the magnetosome [32]. The magnetosome is a lipid vesicle that encapsulates biomineralized magnetite (Fe_3_O_4_) and/or greigite (Fe_3_S_4_, Fig 1C). Dozens of these organelles are organized in chains that allow MMB to sense and orient themselves along Earth’s geomagnetic field in a phenomenon termed magnetotaxis. Magnetosome formation is controlled by a magnetosome gene cluster (MGC, S1 Appendix Text) that encodes several proteins involved in the formation, alignment, and maturation of the organelles [33,34]. The presence of magnetosomes in MMB can be exploited to physically enrich them from environmental samples using a magnet (S1 and S2 Videos). This is particularly important considering that MMB have not yet been successfully cultured and are of low relative abundance (0.001–2%) in their habitats, sulfidic brackish and marine sediments [35–37]. Interestingly, non-magnetotactic multicellular bacteria that affiliate to the same family as MMB (Desulfobacterales) and share many morphological similarities with them have been found in freshwater sediments [38]; these bacteria could be interpreted as MMB that at the time of sampling did not express their magnetosomes or had lost their magnetotactic ability.

While past studies have presented fascinating insights into the cellular organization of MMB and their diverse abilities to sense the environment via light and electron microscopy [22,31,39], their recalcitrance to cultivation has hindered progress towards a better understanding of their physiology and genomics. With the exception of a study that demonstrated chemotactic response of MMB consortia to small molecular weight organic acids [39], questions about their physiology remain unaddressed, and hypotheses about the potential for metabolic differentiation or a division of labor between individual cells within a consortium have not been experimentally tested.

To address these knowledge gaps, we investigated the taxonomic diversity, genomics, physiology, metabolic differentiation, and clonality of MMB inhabiting a tidal pool. To investigate the diversity of MMB within this environment, we sequenced the Single Consortium Metagenomes (SCMs) of 22 individual MMB consortia, representing 8 distinct species of MMB. Comparing the SCMs, we were able to quantify the extent of single-nucleotide polymorphisms (SNPs) between cells composing individual MMB consortia. Our analyses showed that MMB exhibit genetic diversity within a single consortium, indicating that they are not composed of clonal cells. Physiological predictions were established through the reconstruction of species-specific metabolic models. We tested these predictions by performing stable isotope probing (SIP) experiments and analyzing individual consortia using FISH, nanoscale secondary ion mass spectrometry (NanoSIMS), and bioorthogonal noncanonical amino acid tagging (BONCAT). Our results demonstrate that MMB are mixotrophic sulfate reducers and that individual cells within MMB consortia exhibit dramatically different rates of substrate uptake, indicating metabolic differentiation, as well as localized protein synthesis activity.

## Results and discussion

### Genomic features and phylogenetic analysis of MMB

**Fig 2:**
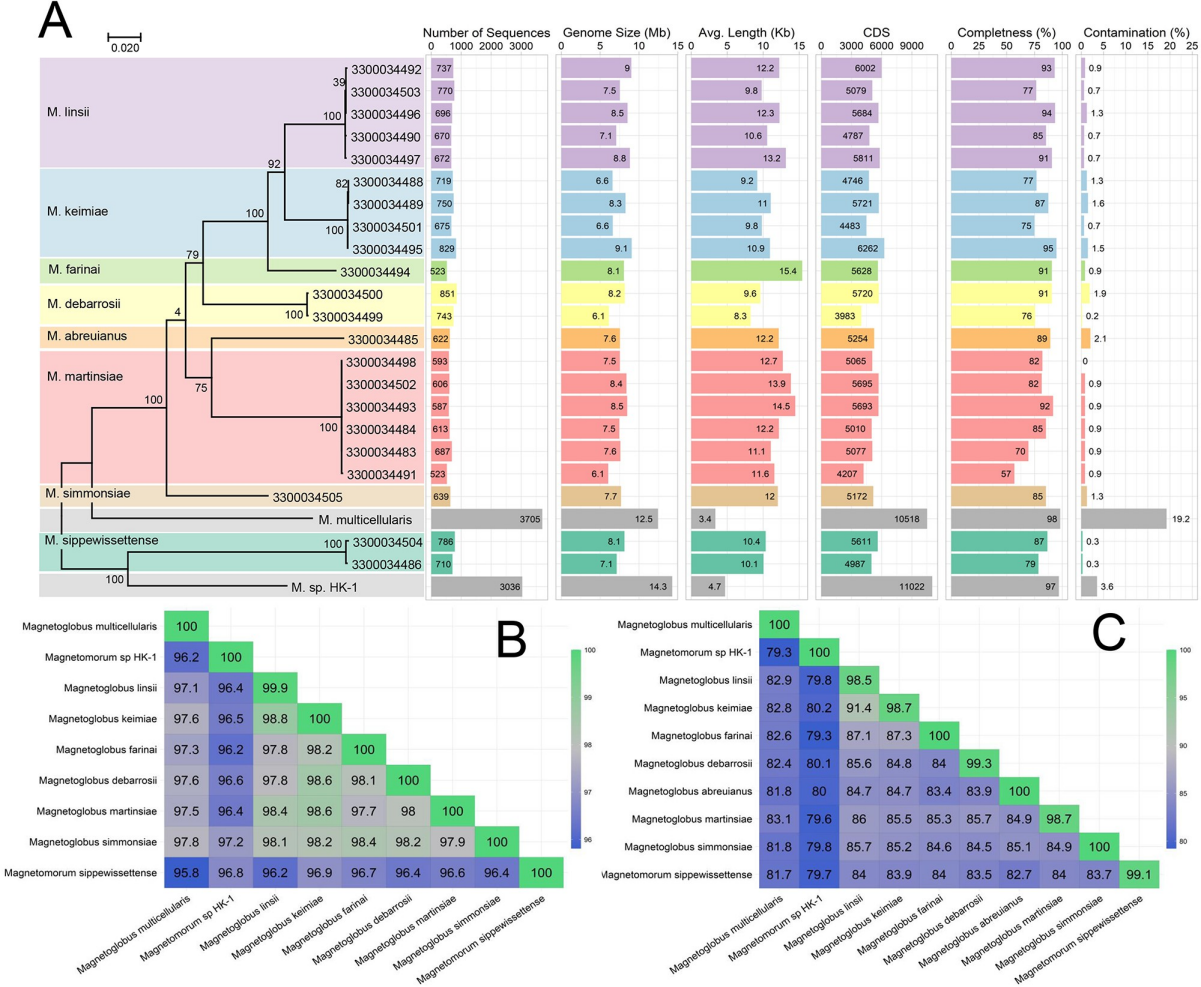
Genomic and phylogenetic analysis of all publicly available MMB MAGs and the 22 SCMs generated in this study. (**A**) Maximum-likelihood tree, inferred with FastTree, using a concatenated set of 6 conserved COGs present in all entries. Ultrafast bootstrap support values and selected genome statistics are listed. The color codes for the SCM Groups remain the same throughout all figures. (**B**) Average full-length 16S rRNA gene identity and (**C**) average genome nucleotide identity heat maps of the 8 newly identified MMB species compared to 2 available MMB reference genomes (*Ca*. M. multicellularis and *Ca*. Magnetomorum sp. HK-1). For a phylogenetic tree of all publicly available MMB 16S rRNA gene sequences, see S2 Fig. For an exhaustive sequence identity analyses of 16S rRNA and whole genomes of MMB, see S3–S5 Figs. The data underlying this figure can be found in Tables A and C in S2 Appendix. MAG, metagenome assembled genome; MMB, multicellular magnetotactic bacteria; SCM, Single Consortium Metagenome.

MMB were recovered from sulfidic sediments collected from a tidal pool in Little Sippewissett Salt Marsh (LSSM; Falmouth, Massachusetts, United States of America; S1A and S1B Fig). This sample site was selected based on the ability to magnetically enrich (S1 and S2 Videos) relatively large quantities of MMB, as previously demonstrated [27,31]. MMB were stained with SYBR green and individual consortia were sorted from a magnetically enriched pellet using fluorescence-activated cell sorting and the DNA of individual sorted MMB was amplified by multiple displacement amplification before Illumina sequencing. From this sample, the SCMs of 22 individual MMB were recovered (Fig 2 and Table A in S2 Appendix). The GC content of the SCMs ranged from 36.2% to 38.4%, which is similar to the GC content observed in previously published MMB draft genomes [22,40,41]. The average and median size of the 22 new SCMs was 7.7 Mb, with a range from 6.1 to 9.1 Mb (Table A in S2 Appendix). Prior to this study, only 3 draft genomes of MMB had been sequenced. These genomes exhibited significant variations in size, ranging from 14.3 Mb for *Ca*. Magnetomorum sp. HK-1 [40], 12.5 Mb for *Ca*. Magnetoglobus multicellularis [22], and 8.5 Mb for MMP XL-1 [41], although the MMP XL-1 genome is not publicly available. The genome sizes of *Ca*. M. multicellularis and *Ca*. M. sp. HK-1 could be conflated due to contamination or the combination of sequence data into the same final bin, as discussed in the respective studies [22,40] and evidenced by our own evaluations of genome contamination (Fig 2A)

Only 14 of the 22 SCMs contained 16S rRNA genes (Table A in S2 Appendix). These sequences, together with publicly available 16S rRNA sequences of MMB as well as those of their single-cell relatives *Desulfosarcina variabilis* and *Ca*. Desulfamplus magnetomortis BW-1, were used to construct a phylogenetic tree (Table B in S2 Appendix). This analysis revealed the presence of 5 phylogenetically distinct genera of MMB in LSSM with high bootstrap support (>75%) (S2 Fig). Analysis of amplicon sequence data obtained in this study and sequences from a previous study at LSSM [27] showed that Group 1 MMB was most abundant in the sample site, constituting 61% of all 16S rRNA genes. Groups 2, 4, 5, and 3 accounted for 21%, 6.5%, 6.5%, and 5% of the 16S rRNA genes, respectively (S2 and S3 Figs).

Phylogenomic analysis of 6 bacterial single copy genes found in all recovered MMB SCMs yielded a topology consistent with the phylogeny derived from the 16S rRNA gene sequences (Figs 2 and S2 and Table C in S2 Appendix). Similarly, whole genome and 16S rRNA-specific average nucleotide identity (ANI) comparison resolved 8 unique species of MMB with >96% ANI. We assigned type genomes for each new MMB species and named them after scientists who have greatly advanced our knowledge of MMB (S1 Appendix and Table D in S2 Appendix).

### Clonality within MMB

MMB have historically been assumed to be clonal due to the synchronized replication of cells during division, which should result in genetically identical daughter cells in the same consortium [15,24]. Additionally, obligate multicellularity has traditionally been thought to perpetuate a clonal population [42]. Although MMB maintain an obligate multicellular life cycle, the degree to which clonality exists within a single consortium has never been experimentally tested. Currently, the only evidence suggesting that cells within MMB are closely related comes from analyses of the 16S rRNA genes from cells of a single genome amplified MMB consortium [40] and a FISH studies demonstrating that cells within individual MMB have identical 16S rRNA sequences [25,27,43,44].

**Fig 3:**
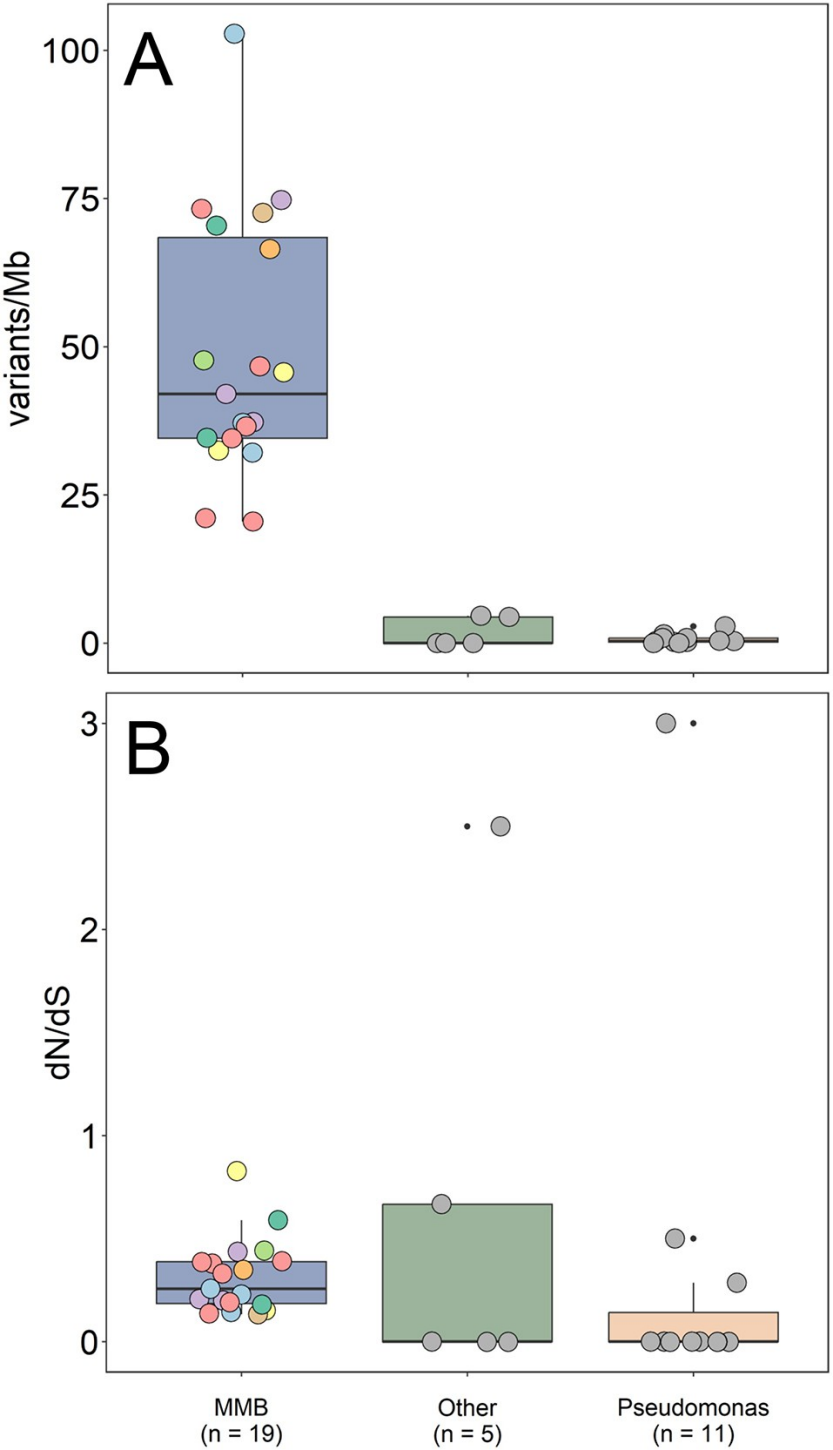
Clonality analysis of individual MMB consortia. (**A**) Individual reads were mapped to the same genome bin for each of the 22 SCMs. This analysis revealed that the genomes of cells within MMB consortia have a higher SNP rate (SNP expressed as variations per Mb) as compared to a clonal *Pseudomonas* sp. control (*p* < 7.3 × 10^−9^, *n* = 10, 30, 60, and 100 *Pseudomonas* cells) and other environmental cells (*p* < 2.4 × 10^−6^, e.g., “Other”). (**B**) The 3 sample categories showed no statistically significant difference in terms of their overall ratio of non-synonymous to synonymous substitutions (dN/dS). Most values were between 0 and 0.5, indicating that there is predominantly strong negative (purifying) selection and that SNPs typically do not lead to changes in the amino acid sequence. S6 Fig provides additional details on genes affected by positive and negative selection. The color of each SCM corresponds to the color identifying each unique species in Fig 2. Statistical analyses were performed using a pairwise *t* test with the Bonferroni *p*-adjusted method. The data underlying this figure can be found in Tables E and F in S2 Appendix. MMB, multicellular magnetotactic bacteria; SCM, Single Consortium Metagenome; SNP, single-nucleotide polymorphism.

We set out to test the hypothesis of clonality using comparative genomics of the 22 MMB SCMs recovered in this study. Reads from each individual SCM were mapped to the corresponding genome bins to quantify SNPs within a single MMB consortium. As a procedural control, 10, 30, 60, and 100 cells of a clonal culture of *Pseudomonas putida* were sorted to construct a mock multicellular consortium. The DNA of MMB consortia and *P*. *putida* controls were amplified using multiple displacement amplification and sequenced using Illumina short read sequencing. Our analysis of the SCMs revealed for the first time that MMB consortia are genomically heterogeneous and thus do not fit the model of clonality for obligate multicellular organisms (Fig 3A). MMB from LSSM contain up to 2 orders of magnitude more SNP differences within a single consortium as compared to the same number of cells from the clonal control (*p* < 7.3 × 10^−9^), with an estimated range of 157 to 789 SNPs in individual SCMs (Fig 3 and Table E in S2 Appendix). Other environmental microbes co-sorted with MMB showed an SNP rate similar to the clonal control and an SNP rate statistically different from the MMB (*p* < 2.4 × 10^−6^), illustrating the uniqueness of MMB. Wielgoss and colleagues performed a similar analysis on fruiting bodies of the aggregative multicellular bacterium *Myxococcus xanthus* in which a comparison of the genomes of cells in fruiting bodies revealed 30 SNP differences between lineages originated from a recent single ancestral genotype [45]. Furthermore, nearly half the mutations detected in the *M*. *xanthus* genomes occurred in the same 6 genes, suggesting there was a strong selection for socially relevant genes, such as a histidine kinase (signal transduction) and methyltransferase (gene expression). Positive selection upon cooperative genes may promote diversity within the organism as a mechanism to increase fitness within spatiotemporally variable environments and protect against social cheaters [46].

To investigate if the genetic heterogeneity within MMB contributes to an increased fitness of the organism, we identified the genes containing SNPs and calculated the corresponding ratio of non-synonymous (dN) to synonymous (dS) substitutions. This analysis showed that the SNP differences within the SCMs of MMB appear to be random with no single gene or category of genes exclusively impacted by the SNPs within or across MMB consortia and that most genes were subject to negative (purifying) selection (Fig 3B and Table F in S2 Appendix). SNPs with a high dN/dS ratio were predominantly found in unannotated genes, such as hypothetical proteins, and were found to be subject to positive selection (S6 Fig). Such genes could ultimately drive functional divergence within the consortium. Other benefits of genomic heterogeneity within MMB are not readily apparent and could be attributed to errors during DNA replication or damaging effects of mutagens. However, it has been shown that a single mutation can lead to a division of labor in bacteria [47]. At this point, it is unclear whether any of the changes we observe in the genomes contained within individual MMB would lead to phenotypic differentiation between the adjacent cells.

### Genome annotation

**Fig 4:**
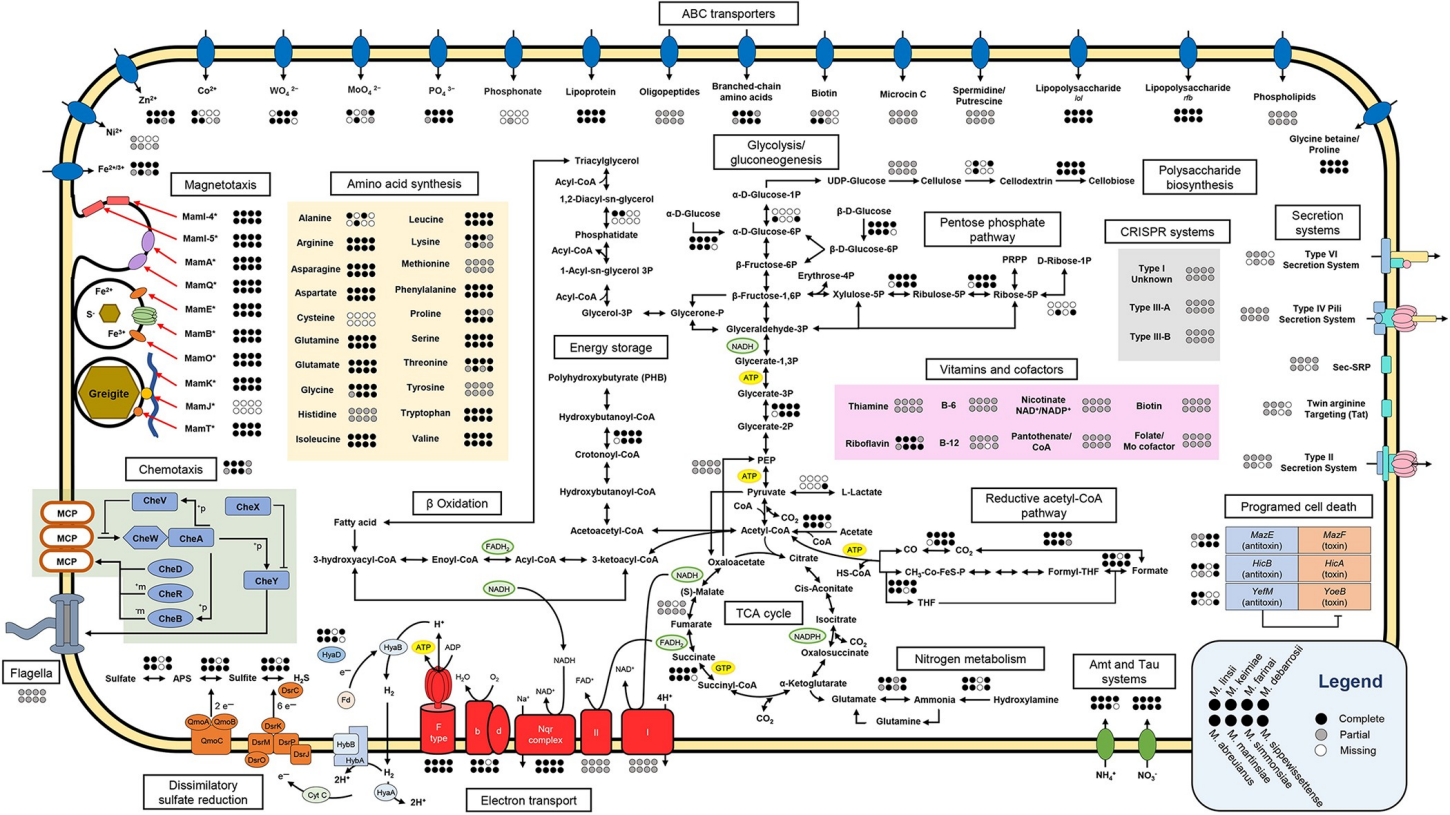
Metabolic potential of the 8 MMB species in LSSM. Arrows without circles indicate presence of the respective enzyme or pathway in all bins. Circles indicate complete presence (black), partial presence (gray), or missing (white) genes in each species. A full list of genes used to construct this figure can be found in Table G in S2 Appendix. LSSM, Little Sippewissett Salt Marsh; MMB, multicellular magnetotactic bacteria.

Metabolic reconstructions of the MMB SCMs (Fig 4 and Table G in S2 Appendix) revealed that all MMB are capable of heterotrophic sulfate reduction and can use acetate, succinate, and propionate as carbon donors and/or electron sources, consistent with previous genomic analyses [22,40]. The SCMs show that LSSM MMB have highly similar metabolic potential. One exception is *Ca*. M. sippewissettense, which lacks the ability to utilize acetyl-coenzyme A (CoA) synthetase and is unable to use acetate, instead likely relying on lactate dehydrogenase to metabolize lactate, a substrate the other species are not capable of using. None of the SCMs contain acetaldehyde dehydrogenase, indicating that MMB are not capable of alcohol fermentation. We resolved a complete glycolysis pathway and TCA cycle as well as reductive CoA pathway in all SCMs. The presence of these genes suggests that MMB in LSSM are capable of both heterotrophic and autotrophic growth using sulfate reduction coupled to hydrogen metabolism, by means of *hyaA/B* and *hybA/B* complexes and oxidative phosphorylation. MMB are genetically capable of shuttling electrons using complexes I, II, and V of the oxidative phosphorylation pathway using F-type ATP synthase complexes, although partial V/A type ATP synthase were found in *Ca*. Magnetoglobus martinsiae and *Ca*. Magnetomorum sippewissettense. In addition, they encode a full Nqr (Na^+^-transporting NADH:ubiquinone oxidoreductase) complex that can move electrons from NADH to ubiquinone with the translocation of a Na^+^ across the membrane. Cytochrome bd oxidase subunits I and II are present in all SCMs, except *Ca*. Magnetoglobus farinai, and could be used to respire molecular oxygen (O_2_) using electrons from cytochrome c or quinols [48]. All species of MMB from LSSM encode rubrerythrin and superoxide reductase, suggesting the possibility that O_2_ could instead be detoxified by the cytochrome bd oxidase (Table G in S2 Appendix) [22,49]. Electrons can also be removed by the reduction of protons to molecular hydrogen (H_2_) by group 1 nickel-iron hydrogenases. The H_2_ can then diffuse across the membrane where HybA/B could oxidize the H_2_, yielding 2 electrons and 2 protons. From there, cytochrome c can shuttle the electrons to the Dsr and Qmo complexes for dissimilatory sulfate reduction.

The MMB SCMs encode several divalent metal transporters, including FoaAB ferrous iron and FepBDC ferric iron transport proteins, indicating they are capable of using both Fe(II) and Fe(III). All SCMs encode phosphate transporters as well as oligopeptide and branched-chain amino acid transporters. Genes for polyamine transport were recovered in the SCMs and may provide resistance to environmental stress such as osmotic pressure and reactive oxygen species [50]. Additionally, each SCM encodes a glycine betaine transporter but does not encode a betaine reductase, indicating that MMB do not use glycine betaine as a nitrogen source but as an osmoprotectant [51]. All MMB species in LSSM, except *Ca*. M. sippewissettense, encode an Amt transporter to transport ammonia into cells that can then be converted into glutamine or glutamate and fed into anabolic pathways. Additionally, each species encodes the NitT/TauT system for nitrate, sulfonate, and bicarbonate transport into cells. The SCMs showed that MMB are capable of synthesizing all canonical amino acids except cysteine and lack cysteine prototrophy genes. Cultures of single-celled magnetotactic bacteria have been found to require the addition of cysteine for growth, suggesting that many magnetotactic bacteria, including MMB, cannot synthesize their own cysteine [52]. The inability to synthesize a sulfurous amino acid is surprising given that most magnetotactic bacteria, including all known MMB, live in sulfur-rich environments.

Previous studies using transmission electron microscopy and Nile Red staining have found large vesicles within MMB cells that have been attributed to carbon/energy or phosphate storage [19,53,54]. Metabolic analysis of the SCMs showed that acetyl-CoA could be condensed and polymerized to polyhydroxybutyrate (PHB) for storage. Furthermore, all necessary genes were identified for *β*-oxidation using triacylglycerol synthesized from the acylation of glycerol-3P with acyl-CoA (Fig 4 and Table G in S2 Appendix). Using Raman microspectroscopy applied to individual MMB, we demonstrated the presence of PHB and lipids, along with Nile Red staining of carbon-rich droplets within cells (S7 Fig and Table H in S2 Appendix). This is, to our knowledge, the first time carbon and energy storage compounds in MMB have been unambiguously identified. Carbon storage has been shown to support the multicellular reproductive life cycles in *Vibrio splendidus* through the specialization of cells during resource limitations [55], suggesting that MMB may utilize a similar mechanism to support their multicellular growth.

Altruistic behavior in biological systems is often favored when relatedness among species is high and the benefit is comparatively large compared to the cost, as has been observed in multicellular myxobacteria [46]. The SCMs revealed that MMB encode *mazE/F*, *hicA/B*, and *yefM/yefB* type II toxin-antitoxin (TA) systems (Fig 4 and Table G in S2 Appendix). TA systems represent an extreme example of altruism in multicellular systems, as individual cells that contribute to the organism by sacrificing themselves through death do not directly benefit from the organism’s multicellularity. But, selection favoring altruistic traits occurs due to the fitness benefits those traits impart on relatives [56]. Detection of CRISPR (clustered regularly interspaced short palindromic repeats) systems I, III-A, and III-B (Table G in S2 Appendix) suggest the TA systems could be used in response to viral infection [57]. The evolution of altruistic cooperation in multicellular organisms has been proposed as a response to environmental stressors [56], indicating the presence of TA systems likely confers increased fitness for MMB in the environment.

### Cell-to-cell adhesion

One of the most intriguing features of MMB is their multicellular life cycle. But how these bacteria maintain their multicellular shape is not entirely known. Previous genomic and microscopic analysis of MMB suggested that exopolysaccharides, adhesion molecules, and Type IV pili could be involved in cell-to-cell adhesion [22,58]. Extracellular matrices, specifically those composed of polysaccharides, have been shown to be important for the development and maintenance of bacterial multicellularity, resulting in several emergent properties that benefit the organism, including the reduction of maintenance energy for individual cells [59]. *Myxobacteria* sp. and *Escherichia coli* have both been shown to use exopolysaccharides to maintain macroscopic biofilms [8,60]. The SCMs recovered in this study encode genes for extracellular polysaccharide biosynthesis, including family-2 glycosyltransferases (GT2), which have been shown to secrete diverse polysaccharides such as cellulose, alginate, and poly-N-acetylglucosamine [61,62]. Specifically, the genes identified in the SCMs were homologous to GT2 Bcs proteins, a bacterial protein complex that synthesizes and secretes a *β*-1,4-glucose polymer (e.g., cellulose) during biofilm formation (Table G in S2 Appendix) [63,64]. The LSSM MMB encode enzymes that catalyze the production of cellulose for biofilm formation (*bcsA*, *bcsQ*, *bcsZ*, *pilZ*, and *bglX*), but lack the co-organization of genes at a single locus as observed for other bacteria [63]. Furthermore, the *bcsB* and *bcsC* subunits were not identified, but additional GT2 as well as *wza* genes that may be involved in the synthesis of exopolysaccharides were present [65]. The catalytic activity of BcsA has been shown to be influenced by the concentration of cyclic dimeric guanosine monophosphate (c-di-GMP) which is in turn affected by environmental oxygen levels [66,67]. Under oxic conditions, the cellular level of c-di-GMP has been shown to increase and bind to BcsA, leading to increased cellulose synthesis [67]. Because MMB commonly exist in oxygen-deficient sediments, cellulose synthesis may be triggered under oxic conditions to stimulate biofilm formation, which has been observed in cultivation attempts of MMB [22].

Filamentous hemagglutinin has been shown to recognize and bind to carbohydrates to facilitate cell-to-cell adhesion in a biofilm [68,69]. The presence of filamentous hemagglutinin genes in our SCMs suggests MMB could use these protein complexes as a mechanism for cell-to-cell adhesion, as previously suggested [22]. Furthermore, the SCMs encode genes for OmpA/F porins, proteins with adhesive properties that have been suggested to interact with exopolysaccharides leading to aggregation of cells [70]. Type IV pili, which have been shown to be involved in cell-to-cell adhesion by interacting with exopolysaccharides [71], were also identified in the SCMs. The pili could alternatively be used for motility, chemotaxis, organization, and DNA uptake [72]. Further investigation into the use of the Type IV pili within MMB is warranted as only predictions can be made from the available genomes.

Previous studies on the membrane of MMB using Ruthenium Red dye and calcium cytochemistry have shown that the consortia are coated in a polysaccharide that extends between cells into the acellular central compartment but the exact composition and structure of this polysaccharide remains unclear [18,58]. Using Raman microspectroscopy, we identified peaks corresponding to exopolysaccharides, confirming the presence of an exopolysaccharide within or surrounding MMB (confocal Raman does not have enough z-resolution to distinguish the in- and out-side of cells; S7 Fig and Table H in S2 Appendix). Cellulase hydrolysis of the MMB resulted in eroded surfaces of the consortia, demonstrating that MMB are indeed covered by a cellulose layer (S8 Fig). Together, these analyses highlight the structural and functional significance of exopolysaccharides required for the multicellular morphotype of MMB.

### Abundance, distribution, and in situ activity of MMB in LSSM

Temporal shifts in MMB groups at LSSM have previously been documented [73] but the abundance of MMB correlated to sediment depth has not yet been analyzed. MMB in the LSSM subsurface were quantified by retrieving a 15-cm core from the tidal pond and determining the fractional abundance of each of the 5 MMB groups recovered throughout the core at centimeter-scale resolution using newly designed FISH probes (S9 Fig and Table I in S2 Appendix). In the top 5 centimeters of sediment, Group 1 MMB accounted for >75% of all MMB while the other groups accounted for 1% to 25%, depending on sediment depth. The total abundance of MMB dropped sharply below 5 cm, where the sediment horizons transitioned from sandy to dense clay sediment containing plant roots. This could be due to MMBs preference for low oxygen conditions, under which sulfate reduction is favored [39,74]. A similar depth-abundance profile was previously observed for the closely related MMB *Ca*. M. multicellularis [74].

BONCAT was used to determine the anabolic activity of MMB Group 1 in the top 6 cm of the LSSM core, which hosted the majority of MMB. Using this approach, we identified a statistically significant difference in MMB activity from 1 cm depth compared to the 2 to 3 cm (*p* < 3.4 × 10^−4^) and from 3 cm compared to 4 to 5 cm (*p* < 3.9 × 10^−3^), below which the MMB population diminished (S10 Fig). The increase of activity of MMB in the first 5 cm of the sediment could be attributed to the circumneutral pH and low redox potential (−260 to −460 mV), as previously observed to be important for the bioavailability of iron and sulfur species for MMB [40].

### Physiology of MMB

**Fig 5:**
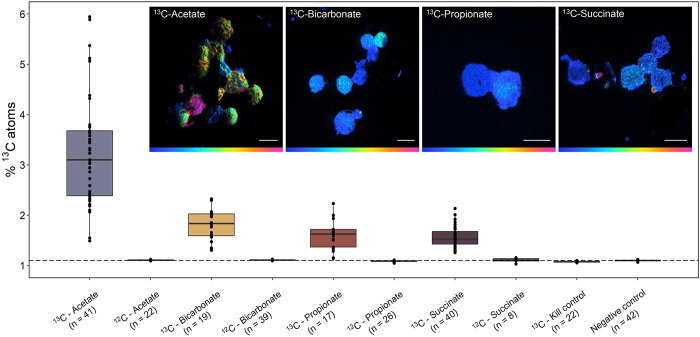
NanoSIMS analysis of the cellular ^13^C-content of MMB consortia after in situ incubation with isotopically light or heavy carbon sources, specifically 1,2-^13^C_2_-acetate, ^13^C-bicarbonate, 1,2-^13^C_2_-propionate, or 1,2-^13^C_2_-succinate, for 24 h. The kill control contained magnetically enriched MMB that had been fixed in 4% paraformaldehyde prior to ^13^C-acetate addition. The negative control was sediment containing MMB without substrate addition. The dotted line shows the natural abundance of ^13^C. For further description of boxplots, see S1 Appendix. Inset images show representative NanoSIMS HSIs for each ^13^C-labeled substrate analyzed. Color scales in HSI images are 1.1%–5% atom percent ^13^C. Scale bars are 5 μm. S1C and S1D Fig show the incubation setup. For a comparison of the anabolic activity of MMB groups 1, 3, and 4, see S11 Fig. S12 Fig provides an example for correlative microscopy analysis of MMB. SI Materials and Methods detail the calculation of atom percent. The data underlying this figure can be found in Table J in S2 Appendix. For ROIs, see S13 Fig. HSI, hue saturated image; MMB, multicellular magnetotactic bacteria; NanoSIMS, nano-scale secondary ion mass spectrometry.

Previous genome- and chemotaxis-based studies suggested that MMB live by heterotrophic sulfate reduction using small organic acids as electron donors [22,39,40]. However, no direct observation of the use of such organics has been reported. Our metabolic reconstructions revealed that all MMB species in LSSM are genetically capable of coupling sulfate reduction to the use of acetate, propionate, and succinate as well as inorganic carbon fixation via the reductive acetyl-CoA pathway. To test whether MMB use these carbon sources to support their growth, we incubated sediment samples with ^13^C-labeled substrates (acetate, bicarbonate, propionate, and succinate) in situ and analyzed individual MMB using NanoSIMS. Consistent with metabolic predictions, MMB took up all ^13^C-labeled substrates (Fig 5 and Table J in S2 Appendix). To identify specific MMB groups, FISH was performed prior to NanoSIMS analyses. Group 1 MMB showed the highest incorporation of ^13^C from acetate as compared to Groups 3 and 4 (*p* < 1.5 × 10^−3^, S11 Fig). We also observed a significant difference between Group 1 and 4 for ^13^C-bicarbonate and ^13^C-propionate uptake (*p* < 3.9 × 10^−3^ and 5.8 × 10^−5^, respectively). At least 3 genera of MMB (i.e., Groups 1–3) assimilated both bicarbonate and propionate (S15 Fig). We were unable to magnetically enrich MMB from a sediment sample incubated with ^13^C-acetate and molybdate, an inhibitor of sulfate reduction, indirectly demonstrating that MMB are in fact sulfate reducers. In summary, our analyses demonstrated that LSSM MMB are capable of assimilating both inorganic and organic carbon, indicating autotrophic and heterotrophic growth, and that different groups of MMB demonstrate variable affinities for carbon sources.

### Metabolic differentiation as studied by SIP-NanoSIMS

**Fig 6:**
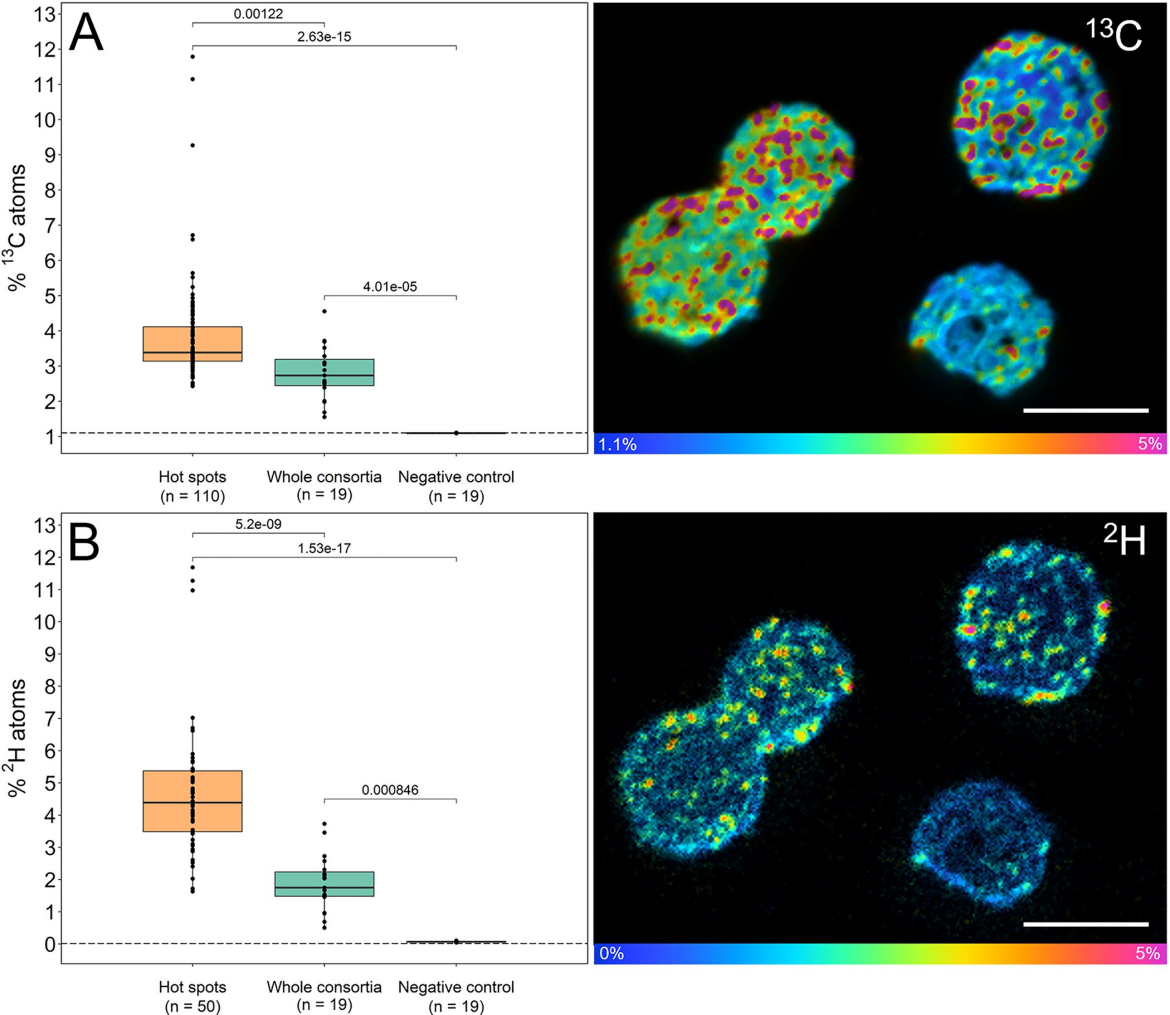
NanoSIMS analysis of MMB consortia incubated with 1,2-^13^C_2_-acetate and ^2^H_2_O. To avoid human bias, ROIs for hotspots within individual consortia were auto-segmented in ImageJ and the isotope ratios of hotspots compared to the value for the whole consortium and negative controls. The ^13^C and ^2^H hotspots showed significantly higher isotopic enrichment when compared to the values for the respective whole consortium (*p* < 1.3 × 10^−3^ and <5.2 × 10^−9^, respectively), indicating they are metabolically differentiated. For further description of boxplots, see S1 Appendix. NanoSIMS HSI images of the same MMB consortia analyzed using mass ratio ^13^C^12^C/^12^C_2_ and ^2^H/^1^H, revealing cell-to-cell differentiation. The HSI are scaled to show the atom percent of the respective isotope. Scale bars in HSI images equal 5 μm. For an example of the correlative microscopy workflow used to study MMB, see S12 Fig. The data underlying this figure can be found in Table K in S2 Appendix. For ROIs of all consortia, see S14 Fig. Statistical analyses were performed using a pairwise *t* test with the Bonferroni *p*-adjusted method. HSI, hue saturated image; MMB, multicellular magnetotactic bacteria; NanoSIMS, nano-scale secondary ion mass spectrometry.

A hallmark of multicellularity is the existence of a division of labor [6,12]; however, because of their recalcitrance to cultivation, this hypothesis has never been addressed in MMB. Lacking a culture of MMB and established molecular techniques, such as mRNA-FISH, makes confirming a division of labor within this organism’s consortium challenging. To address the existence of a division of labor within MMB, we investigated whether consortium members are metabolically differentiated by performing in vitro incubations of a magnetic enrichment of MMB with ^13^C-labeled acetate and deuterium oxide (^2^H_2_O), as cellular labeling from the latter is a general proxy for metabolic activity [75]. Samples analyzed using NanoSIMS showed variation of isotopic signal across cells within individual consortia, indicating different metabolic activity within MMB (Fig 6 and Table K in S2 Appendix). The mass ratio for each isotope label was quantified and areas of high anabolism (referred to as “hotspots”) within the consortium compared to the value of the same isotope label for the whole consortium. This analysis demonstrated a statistically significant difference of anabolic activity between hotspots and the whole consortium for both ^13^C and ^2^H_2_O (*p* < 1.3 × 10^−3^ and <5.2 × 10^−9^, respectively). Comparison of SEM and NanoSIMS imaging shows that the extent of SIP labeling varies between single cells as well as across the entire MMB consortium (S12 Fig). The hotspots do not exhibit localization in any specific region of an MMB. However, they are not uniformly distributed throughout the consortium, demonstrating variations in metabolic activity with some areas displaying lower metabolic activity than others. To further investigate the localization of the isotope within the individual consortium, we applied a median filter ratio to the hue saturated images (HSIs) using different kernel radii [76]. This method averages the isotopic ratio over the given pixel radius, revealing sub-consortium localization across the MMB (S15 Fig). Together, our analyses show that metabolism of ^13^C-acetate and ^2^H-water is not uniform across the MMB, suggesting a differentiation in metabolic activity within individual consortia. Similar differences in the uptake of isotope-labeled substrate have also been reported for cellularly and metabolically differentiated cells of filamentous cyanobacterium *Anabaena oscillarioides* [77].

### Metabolic differentiation as studied by BONCAT

**Fig 7:**
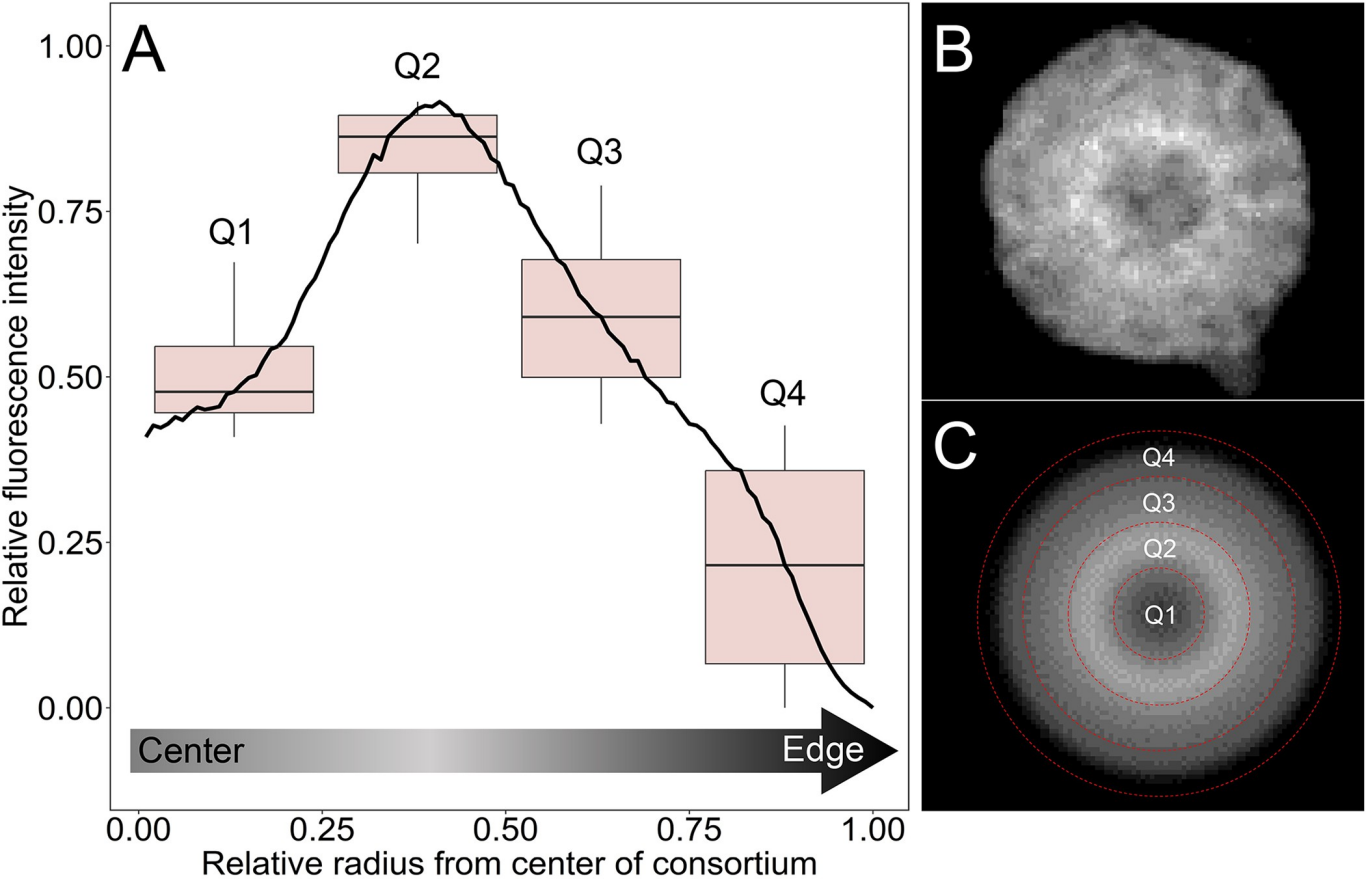
Heterogeneity in anabolic activity within individual MMB consortia as revealed by BONCAT. (**A**) The averaged intensity profile across the diameter of 57 rotationally averaged BONCAT-labeled MMB with standard deviation shown in gray. RFI and diameter of each MMB was scaled as a ratio (0 to 1) to account for differences in fluorescence intensity between consortia and size of consortia. The boxplots show the averaged RFI for each quarter section of the radius with a pairwise statistical difference of *p* < 1.0 × 10^−10^. For further description of boxplots, see S1 Appendix. (**B**) Gray scale confocal microscopy image of a BONCAT-labeled MMB showing proteins that had been synthesized over a 24-h period. (**C**) Image of the MMB shown in (**B**) that has been rotationally averaged prior to quantification in Eman2. The red dotted line shows each quarter analyzed for the boxplots shown in (**A**). For raw and rotationally averaged images of all 57 MMB, see S16 Fig. Statistical analyses were performed using a pairwise *t* test with the Bonferroni *p*-adjusted method. The data underlying this figure can be found in Table L in S2 Appendix. BONCAT, bioorthogonal noncanonical amino acid tagging; MMB, multicellular magnetotactic bacteria; RFI, relative fluorescence intensity.

To determine if protein synthesis was localized to specific or individual cells within the consortium, we combined BONCAT with confocal laser scanning microscopy. Our analysis revealed an apparent gradient of newly synthesized proteins within each cell of the consortium, showing localization around the acellular center of individual consortia (Fig 7). This distinct pattern of protein synthesis was observed in all 57 MMB examined (S16 Fig) with negative controls showing no fluorescence (S17 Fig). The localization of newly synthesized protein around the acellular center of the consortium suggests this area is highly active; however, the reason is currently unknown. Cells within the consortium could engage in a division of labor by metabolizing specific substrates (e.g., acetate) and then sharing those resources with other cells through the acellular space, possibly by the utilization of membrane vesicles [58]. A prime example of a division of labor in multicellular bacteria is the filamentous cyanobacteria *Anabaena*. This organism has established a mutually beneficial interaction between the heterocyst and vegetative cells via intercellular exchange of metabolites through septal junctions [6,78]. However, there is no evidence that such pores or channels exist in MMB, although an alternative route for metabolite transfer could be the acellular space within the consortium. This space has been hypothesized to be used for communication and metabolite exchange because it provides the shortest distance between any 2 cells [58]. The localization of newly synthesized protein around the acellular center of the consortium suggests this area is highly active, possibly for exchange of metabolites from cells that are hotspots for anabolic activity. This implies cells within the consortium could metabolize specific substrates (e.g., acetate) and then share those resources with other cells through the acellular space, possibly by the utilization of membrane vesicles [58].

## Conclusions

In summary, our study demonstrated that cutting-edge culture-independent approaches can reveal fundamental biology of yet uncultured multicellular microorganisms. We showed that MMB exhibit a higher level of complexity than previously thought by maintaining genomic heterogeneity and metabolic differentiation among the individual cells of a consortium. Moreover, we provided a detailed analysis of the genetic potential of 8 newly discovered species of MMB as well as their ecology, ecophysiology, and in situ activity. We hope that these results will eventually lead to MMB representatives being brought into culture. In addition, our results provide the basis for future experiments to further explore the mechanisms of cell-to-cell heterogeneity. Specifically, we expect mRNA-FISH [79,80] studies to reveal to what extent gene expression levels differ from cell to cell, and SIP-NanoSIMS and spatial metabolomics [81] to reveal the molecular underpinnings of cellular interactions. Modeling approaches could shed light on potential metabolic networks within the consortium, further supporting the hypothesis of a division of labor. Additionally, such approaches could enable a deeper understanding of the propagation and constraints on genomic diversity observed within individual MMB consortia. Given that the biology of MMB is, as far as we know, unique in the bacterial domain, we propose MMB should, despite their recalcitrance to cultivation, receive higher attention by researchers interested in the evolution and biology of bacterial multicellularity.

## Materials and methods

### MMB sorting, single consortia genomic sequencing, and clonality analyses

A sediment sample from LSSM was shipped overnight to the Joint Genome Institute (JGI, then Walnut Creek, California, USA) where a magnetic enrichment was performed to obtain a pellet of MMB (see S1 Appendix Methods for details). The sample was stained with the DNA-stain SYBR Green (Thermo Fisher, Eugene, Oregon, USA) and large, fluorescent particles sorted using a BD Influx fluorescence-activated cell sorter (448 nm excitation of SYBR versus side scatter; S18 Fig) to obtain individual MMB consortia in single wells of a 384-well plate. In addition, replicates of 10, 30, 60, and 100 cells from a culture of *Pseudomonas putida* KT2440 that had been grown in LB media were sorted into single wells as a mock control for clonal multicellularity. The *P*. *putida* culture liquid culture was initiated from a single colony picked from an LB agar plate. Sorted MMB and *P*. *putida* were then lysed and DNA amplified via the WGA-X protocol [82]. Amplified SCMs were screened using 16S rRNA gene PCR according to DOE JGI standard protocols [83]. Next, sequencing libraries were generated from amplified DNA using the Nextera XT v2 library preparation kit (Illumina) and sequenced on the Illumina NextSeq platform. Assemblies were derived from the IMG/M database [84]. Contigs larger than 2 kb were organized into genome bins based on tetranucleotide sequence composition with MetaBat2 [85] with default settings. Metagenome assembled genome (MAG) completeness and contamination were estimated with CheckM (v1.012) [86]. Gene calling was performed with Prodigal [87] using the bacterial code (translation table 11). Average nucleotide identities (ANI) between MAGs were calculated with FastANI (v1.1) [88], filtered at 95% sequence identity and 30% aligned fraction, and then clustered using mcl (v14-137) [89].

We assessed clonality of sorted MMBs, single sorted and amplified *Pseudomonas* controls and other MAGs derived from sorted MMBs by mapping the reads from the respective libraries to the contigs larger than 5 kb in assemblies derived from the same library using BBMap (v38.79) (https://sourceforge.net/projects/bbmap/, [90]) with the flags minid = 0.95 minaveragequality = 30. Variants were called with the BBTools script callvariants.sh using the flags minreads = 2 minquality = 30 minscore = 30 minavgmapq = 20 minallelefraction = 0.05 and identified variants were then annotated as synonymous (s), nonsynonymous (ns), or intergenic depending on their position. Variants made up by one or more Ns were excluded from the analysis. To investigate differences between MMB, all libraries were also mapped to contigs with a size of at least 5 kb from the longest MMB assembly (3300034493).

### Stable isotope probing

To empirically test the use of carbon substrates as predicted by the functional annotation of MMB SCMs and determine the anabolic activity of MMB cells, we performed both in situ and in vitro incubations of MMB with ^13^C- and ^2^H-labeled substrates (all 99.9%, Cambridge Isotopes Laboratories). The in situ incubations were performed in duplicate on August 28, 2022 at LSSM by amending 200 ml top sediment slurry with 2 mM ^13^C-1,2-acetate, 2 mM ^13^C-1,2-succinate, 5 mM ^13^C-1,2-propionate, 5 mM ^13^C-bicarbonate, or 2 mM ^13^C-1,2-acetate plus 8 mM molybdate (a competitive inhibition of sulfate reduction). A negative control to which no amendment was made as well as a killed control in which biomass had been preincubated with 4% paraformaldehyde (PFA) for 60 min at ambient temperature prior to addition of 2 mM ^13^C-1,2-acetate were also performed. Samples were stored in 200 ml Pyrex glass bottles (Corning, Glendale, Arizona, USA) and incubated for 24 h in situ at the sample site where they were buried 4 to 6 cm below the sediment in a basket (S1C and S1D Fig). The in vitro incubations were performed by incubating magnetically enriched MMB in 10 ml of 0.22 μm filter sterilized (Millipore, Burlington, Massachusetts, USA) LSSM water amended with the same amendments as the in situ incubations, as well as 50% deuterium oxide (D_2_O), for 24 h at ambient lab temperature (approximately 23°C) in the dark. At the end of each incubation period, MMB were magnetically enriched and fixed with 4% PFA for 60 min at ambient temperature. Cells were centrifuged for 5 min at 16,000 g, after which the supernatant was removed, and the cell pellets resuspended in 50 μl 1× PBS and stored at 4°C.

### NanoSIMS

Samples were prepared for NanoSIMS on stainless steel coupons as previously described [91]; for details, see SI Materials and Methods. To quantify cell-to-cell differences in isotope uptake within individual consortia, ROIs were selected around localized densities (i.e., hotspots) of masses corresponding to the respective substrate and compared to whole consortia values for the same isotope of interest. In order to not introduce human bias, ROIs were selected in Fiji (https://imagej.net/software/fiji/) by converting the mass image to an 8-bit image for which the brightness and contrast adjusted to help identify the localized densities for the mass of interest (e.g., ^12^C^2^H 14.02, ^12^C^13^C 25.00) using the thresholding functions within the software.

### BONCAT

BONCAT was performed as previously described [92]; for details, see SI Materials and Methods. To evaluate cell–cell differences in anabolic activity of individual consortia, MMB were imaged by taking z-stacks (approximately 300 nm per image) of the entire consortia using an Inverted DMI8 Stellaris 8 Confocal Microscope (Leica Microsystems). Images focused on the center of the consortia were selected and Eman2 [93] was used to select individual MMB for particle analysis. Each image was then filtered using an edge mean normalization, center of mass xform, and rotational average math settings (S16 Fig). Because of varying sizes of consortia, a Python script was used to determine the radius of each consortium by calculating the number of pixels from the center of mass, as determined by the filter, to where the standard deviation of the pixels is <0.01. The radius of all consortia was standardized by dividing 1 by the radius. Additionally, the average fluorescence intensity was normalized by calculating $I_{norm}=\frac{I_{ori}-I_{min}}{I_{max}-I_{min}}$, where *I*_*ori*_ is the original fluorescence intensity value and *I*_*min*_*/I*_*max*_ are the minimum and maximum relative fluorescence intensity (RFI) values for the individual consortia. The average and standard deviation of data was calculated and plotted using R. All code used for analysis is deposited on GitHub (https://github.com/georgeschaible/MMB-BONCAT).

### Supplementary methodology

Sample collection, phylogenetic analysis, genome and magnetosome analyses, FISH, BONCAT, NanoSIMS, Raman microspectroscopy, and SEM experiments, geochemical analysis, and statistical analyses are described in the SI Materials and Methods.

## Supporting information

S1 Fig Little Sippewissett salt marsh, Falmouth MA. (**A**) Photo of the tidal pool from which sulfidic sediments were obtained, facing west towards Buzzards Bay. (**B**) Map of the salt marsh showing the tidal pool in red and water in white. (**C**) Each sample was incubated in a 200 ml bottle filled to the top with the sediment slurry and tightly capped. Because no MMB could be recovered post-fixation from the kill control sample, 200 μl of sample were incubated in a small glass vial inside of the 200 ml bottle. (**D**) Samples were incubated in situ below the sediment at the site for 24 h. All photos were taken by George A. Schaible. Base layer of map made using USGS topographical map (https://www.sciencebase.gov/catalog/item/5b3cb9eee4b060350a0a9ae2).
(PDF)

S2 Fig Phylogenetic analysis of MMB using near-full length 16S rRNA genes (length listed next to name) found in 14 of the 22 SCMs and in reference genomes. Tree reconstructed using maximum likelihood method with bootstrap values calculated using 500 replicates. Bootstrap values above 50 are shown. *Ca*. M. abreuianus is not shown in this analysis because no 16S rRNA gene was recovered from the SCM. Color coded sequences belong to their respective SCM, as shown in Table A in S2 Appendix. Bars on right show specificities of our newly designed FISH probes that target genus-level groups of MMB in LSSM (Table I in S2 Appendix).
(PDF)

S3 Fig Near-full length 16S rRNA gene comparison of all sequences recovered in this study and previous studies at LSSM (Simmons and Edwards, 2007). Percent identity values are shown within boxes. Bars on left highlight MMB groups for which genus-level FISH probes designed (Table I in S2 Appendix).
(PDF)

S4 Fig Near-full length 16S rRNA identity comparison for the 14 sequences recovered from SCMs and the 2 MMB reference genomes (*Ca*. M. multicellularis and *Ca*. Magnetomorum sp. HK-1). Percent identity values are shown within boxes.
(PDF)

S5 Fig Genome ANI comparing each of the 22 individual SCMs with the 2 publicly available reference genomes (*Ca*. M. multicellularis and *Ca*. Magnetomorum sp. HK-1). ANI values are shown within boxes.
(PDF)

S6 Fig Heatmap and cluster analysis of pfams annotation of individual SNPs showing the log2 ratio of non-synonymous to synonymous substitutions (dN/dS) for the SNP differences contained within each SCM. The analysis suggested that most genes were subject to negative (purifying) selection. On the other hand, genes without functional annotation (“no hits”) were frequently subject to positive selection. The data underlying this figure can be found in Table F in S2 Appendix.
(PDF)

S7 Fig Representative Raman spectrum of an MMB using a 532 nm laser. Vertical lines show peaks corresponding to polyhydroxybutyrate (blue), triglycerides (gold), and exopolysaccharides (pink). Wavenumbers corresponding to peaks are listed in Table F in S2 Appendix. The large peak at approximately 335 cm^−1^ is assigned to the magnetosome crystal greigite, which has previously been shown for MMB from the same site (Schaible and colleagues). Inset image shows an MMB consortium stained with Nile Red, indicating C-H rich droplets within cells. The contrast and brightness of the image has been increased for better visualization. Scale bar is 5 μm.
(PDF)

S8 Fig Cellulase treatment of MMB. (**A1-3**) Control sample of MMB incubated without cellulase. (**B1-3**) After treatment with cellulase the surface of MMB consortia was noticeably eroded as compared to the control. Both samples were incubated for 1 h under otherwise identical conditions (pH, temperature, and osmolarity). All scale bars are 1 μm.
(PDF)

S9 Fig Fractional abundance of MMB groups by depth in LSSM. (**A**) Image of the 15 cm core taken from the West end of sampling site prior to being sectioned into 1 cm horizons from which MMB were enriched for quantification by FISH. (**B**) DOPE-FISH analysis of MMB Groups 2 (red) and 5 (green) shown in panel (**B**) and Groups 1 (green), 3 (yellow), and 4 (red) shown in panel (**C**). MMB not detected by the respective FISH probes are shown in the blue DAPI counterstain in the microscopy images. Scale bars are 5 μm. Bar plots show the abundance of each MMB group as determined by DOPE-FISH for each centimeter of the sediment core shown in panel (**A**). Unlabeled populations are MMB that were stained with DAPI but were not detected by the FISH probes used in the 2 separate experiments and are shown in gray. Consistent with results from SCM and previous 16S rRNA gene abundance studies (Simmons and Edwards, 2007) in LSSM, Group 1 numerically dominate the MMB population. FISH probes used in this experiment are detailed in Table I in S2 Appendix. Statistical analyses were performed using a pairwise *t* test with the Bonferroni *p*-adjusted method. The data underlying this figure can be found in Table P in S2 Appendix. Photo by George Schaible.
(PDF)

S10 Fig Anabolic activity of MMB inhabiting the top 6 cm of LSSM sediment as measured by BONCAT. (**A**) 1 cm sediment core horizons were incubated in the presence of the methionine analogue HPG and magnetically enriched MMB stained via azide-alkyne click chemistry with Alexa Fluor 405 to show relative activity of Group 1 MMB as a factor of depth in the sediment. The vertical line within each box shows the median and the whisker shows the range of the data. Dots represent individual MMB that were measured and analyzed using the software package Daime. Data points that were more than 2 standard deviations of the mean are shown as individual points past the whicker. The analysis showed that there is a statistically relevant difference in the activity of MMB from 1 cm depth to 2 to 3 cm and again from 2 to 3 cm to the 4 to 5 cm depth. (**B**) Exemplary epifluorescence microscopy image of click-stained MMB. (**C**) Overlay epifluorescence microscopy image of FISH-labeled MMB shown in panel B. Group 1 is shown in green, Group 3 in yellow, and Group 4 in red. All scale bars are 5 μm. All statistically differences are shown: ** = *p* < 3.9 × 10–3, *** = *p* < 3.5 × 10–4. FISH probes used in this experiment are detailed in Table I in S2 Appendix. Statistical analyses were performed using a pairwise *t* test with the Bonferroni *p*-adjusted method. The data underlying this figure can be found in Table Q in S2 Appendix.
(PDF)

S11 Fig Comparison of ^13^C-labeled substrate incorporation by MMB Groups 1, 3, and 4 using NanoSIMS analysis of mass ratio ^13^C^12^C/^12^C_2_. The analysis shows that MMB in Group 1 anabolize acetate at a statistically greater rate than Groups 3 and 4 (*p* < 8.9 × 10^−3^). Group 1 also incorporated more bicarbonate than Group 4 (*p* < 2.4 × 10^−2^), although Group 4 only contained 4 samples to compare. Statistical analyses were performed using a pairwise *t* test with the Bonferroni *p*-adjusted method. The data underlying this figure can be found in Table K in S2 Appendix.
(PDF)

S12 Fig Correlative imaging of MMB. The (**A**) taxonomy (DOPE-FISH), (**B**) morphology (SEM), (**C**) distribution of sulfur (NanoSIMS, mass 32; a proxy for the presence of sulfur-containing magnetosomes), and (**D**) uptake of 1,2-^13^C_2_-labeled acetate (NanoSIMS, HSI image showing mass ratio ^13^C^12^C/^12^C_2_). Scale bars are 5 μm. The HSI mass ratio color scale in (**D**) is 1.1%–5% atom percent.
(PDF)

S13 Fig ROIs for NanoSIMS substrate analysis shown in Fig 5 of main text. Because the in situ incubation incurred particles that were not of interest (e.g., diatoms and particulates), the ROIs were hand drawn around each MMB using the mass 26.00 (^12^C^14^N) channel to avoid incorporation of exogenous material in the analysis. (**A1**) ^13^C-acetate, (**A2**) ^12^C-acetate, (**B1**) ^13^C-bicarbonate, (**B2**) ^12^C-bicarbonate, (**C1**) ^13^C-propionate, (**C2**) ^12^C-propionate, (**D1**) ^13^C- succinate, (**D2**) ^12^C-succinate, (**E**) ^13^C-acetate kill control, (**F**) negative control. ROIs are shown in green and red outlines.
(PDF)

S14 Fig ROIs for NanoSIMS hotspot analysis shown in Fig 6 of main text. As to avoid introducing bias into the selection of hotspot ROIs, thresholding in ImageJ was used to automatically select for ROIs, as outlined in the methods. The respective mass image was used for hotspot thresholding and ROI selection. ROIs for whole consortia were hand drawn. All ROIs are show in red outlines.
(PDF)

S15 Fig Median filter ratio radius effect on HSI NanoSIMS images of ^13^C and ^2^H hotspots (**A–C**) Mass ratio (^2^H^12^C/^1^H^12^C) of MMB labeled with deuterium oxide (^2^H_2_O). (**D–F**) Mass ratio (^13^C^12^C/^12^C_2_) of the same MMB shown in (**A–C**) but labeled with 1,2-^13^C_2_-labeled acetate. For these images, the median filter ratio radius was increased to show the effect of noise reduction and localization of isotope label within consortia. A higher filter radius reveals isolated areas of the respective isotope label within MMB, though for a radius >5, the label is averaged over an area greater than the size of a single cell within the consortium, thus losing cellular resolution. Independent of the radius chosen, hot spots remain visible.
(PDF)

S16 Fig Anabolic activity within individual consortia. (**A**) Gray-scale images of individual MMB stained via azide-alkyne click chemistry with Alexa Fluor 488. (**B**) The same consortia shown in (**A**) that have been rotationally averaged in Eman2 software. The relative fluorescence intensity was standardized for all samples prior to analysis.
(PDF)

S17 Fig Controls for metabolic differentiation as studied by BONCAT. (**A**) AHA positive BONCAT *E*. *coli* control. (**B**) AHA negative BONCAT *E*. *coli* control. (**C**) AHA positive BONCAT of MMB. (**D**) AHA negative BONCAT of MMB. (**E**) The exposure time was adjusted to allow for visualization of the AHA negative MMB from panel (**D**), which resulted in a dramatic over exposure (**F**) of the AHA positive MMB from panel (**C**).
(PDF)

S18 Fig Fluorescence activated cell sorting of a magnetically enriched sample from tidal pond sediment stained with SYBR Green. A sorting gate, presumed to contain MMB consortia, was set around particles with a strong 488 nm signal and high side scatter (SSC), indicating a large cell size. Other particles likely are single-cell magnetotactic bacteria or non-magnetotactic bacteria present in the pond water. MMB consortia were sorted into individual wells of a microtiter well plate and 22 MMB consortia were genome sequenced.
(PDF)

S19 Fig Gene synteny for scaffolds containing the magnetosome gene clusters compared. The corresponding annotations of colored genes are shown in the legend to the right.
(PDF)

S1 Appendix Supporting Results, Discussion, and Materials and Methods. (DOCX)

S2 Appendix Supplementary tables A–Q. **Table A in S2 Appendix.** Genome features of SCM recovered from LSSM MMB as well as available reference genomes. **Table B in S2 Appendix.** List of accession numbers used for 16S rRNA phylogenetic analysis. **Table C in S2 Appendix.** Identification of common single copy genes across all SCMs and reference genomes used to make phylogenomic tree. Genes shown in green were used to construct the phylogenetic tree in Fig 2. **Table D in S2 Appendix.** List of researchers and location as well as etymologies for MMB species. **Table E in S2 Appendix.** Analysis of clonality within individual MMB. **Table F in S2 Appendix.** Analysis of synonymous verses non-synonymous mutations within SCMs. **Table G in S2 Appendix.** Metabolic pathway and functional annotations for SCMs from IMG annotations. Absence of gene in a specific SCM is denoted by a—symbol. Locus tag is listed for each gene present in SCM. **Table H in S2 Appendix.** Raman wavenumbers corresponding to biomolecules. **Table I in S2 Appendix.** List of FISH probes. All probes bind at positions 1032–1049 (using *E*. *coli* 16S rRNA as reference) and compete with each other for their binding site. See S9 Fig, S10 Fig, and S12 Fig for examples of FISH. **Table J in S2 Appendix.** NanoSIMS data for in situ experiments showing taxonomy via FISH as well as substrate amendments. **Table K in S2 Appendix.** NanoSIMS data for in vitro experiments comparing hotspots to whole consortium and controls. **Table L in S2 Appendix.** Relative radii and fluorescence intensity for BONCAT showing heterogeneity in anabolic activity within individual MMB consortia. Values determined using Eman2. **Table M in S2 Appendix.** Media recipe for cultivation of MMB. **Table N in S2 Appendix.** Water chemistry of LSSM sample site at 2 time points. Water collected above sediment during low tide. **Table O in S2 Appendix.** Comparative analysis of magnetosome genes using a BLAST of *Candidatus* Desulfamplus magnetomortis BW-1 magnetosome gene cluster genes against each SCM and the available reference genomes. **Table P in S2 Appendix**. Fractional abundance data for MMB groups analyzed by depth in LSSM for S9 Fig. Count adjusted (Corrected Count) for volume of original sample. **Table Q in S2 Appendix**. Relative fluorescence intensity calculations for BONCAT experiment shown in S10 Fig.
(XLSX)

S1 Video Third and final round of a magnetic enrichment of MMB. MMB swim from the bottom of the microcentrifuge tube up towards the magnetic North of a magnetic stir bar. Video is 30× the original speed.
(MOV)

S2 Video MMB swimming at the edge of a hanging water droplet towards the magnetic North of a magnetic stir bar that is out of frame. The temporal response of MMB to changes in the magnetic field is observed when the magnet is turned. This switches the magnetic field and induces a change in the swimming direction of the MMB consortia, until the magnet is turned again, and the MMB swim back to the edge of the hanging water droplet. Video is at its original speed.
(MOV)

## Abbreviations

ANI: average nucleotide identity
BONCAT: bioorthogonal noncanonical amino acid tagging
FISH: fluorescence in situ hybridization
HSI: hue saturated image
LSSM: Little Sippewissett Salt Marsh
MAG: metagenome assembled genome
MGC: magnetosome gene cluster
MMB: multicellular magnetotactic bacteria
NanoSIMS: nano-scale secondary ion mass spectrometry
RFI: relative fluorescence intensity
SCM: Single Consortium Metagenome
SEM: scanning electron microscopy
SIP: stable isotope probing
SNP: single-nucleotide polymorphism
TA: toxin-antitoxin
