# Supplementary material for: Multicellular magnetotactic bacteria are genetically heterogeneous consortia with metabolically differentiated cells
Source: PLoS Biol. 2024 Jul 11;22(7):e3002638. doi: 10.1371/journal.pbio.3002638 (PMC11239054; doi:10.1371/journal.pbio.3002638)
Supplement: S1 Fig — (A) Photo of the tidal pool from which sulfidic sediments were obtained, facing west towards Buzzards Bay. (B) Map of the salt marsh showing the tidal pool in red and water in white. (C) Each sample was incubated in a 200 ml bottle filled to the top with the sediment slurry and tightly capped. Because no MMB could be recovered post-fixation from the kill control sample, 200 μl of sample were incubated in a small glass vial inside of the 200 ml bottle. (D) Samples were incubated in situ below the sediment at the site for 24 h. All photos were taken by George A. Schaible. Base layer of map made using USGS topographical map (https://www.sciencebase.gov/catalog/item/5b3cb9eee4b060350a0a9ae2). (PDF) [file pbio.3002638.s001.pdf]

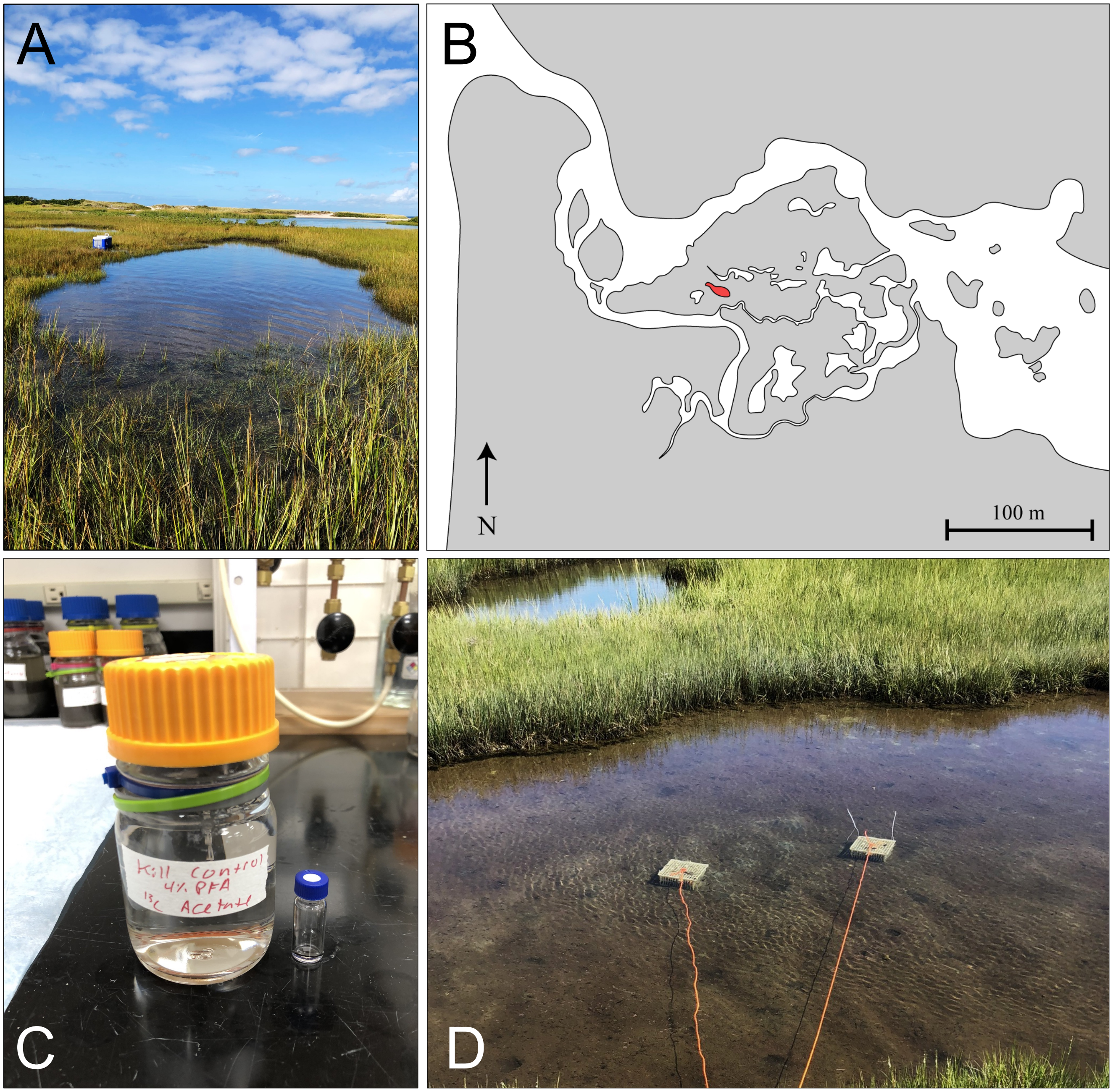

Fig. S1. Little Sippewissett salt marsh, Falmouth MA. (**A**) Photo of the tidal pool from which sulfidic sediments were obtained, facing west towards Buzzards Bay. (**B**) Map of the salt marsh showing the tidal pool in red and water in white. (**C**) Each sample was incubated in a 200 mL bottle filled to the top with the sediment slurry and tightly capped. Because no MMB could be recovered post-fixation from the kill control sample, 200 µL of sample were incubated in a small glass vial inside of the 200 mL bottle. (**D**) Samples were incubated *in situ* below the sediment at the site for 24 hours. All photos were taken by George Schaible.
