# Supplementary material for: Multicellular magnetotactic bacteria are genetically heterogeneous consortia with metabolically differentiated cells
Source: PLoS Biol. 2024 Jul 11;22(7):e3002638. doi: 10.1371/journal.pbio.3002638 (PMC11239054; doi:10.1371/journal.pbio.3002638)
Supplement: S5 Fig — ANI values are shown within boxes. (PDF) [file pbio.3002638.s005.pdf]

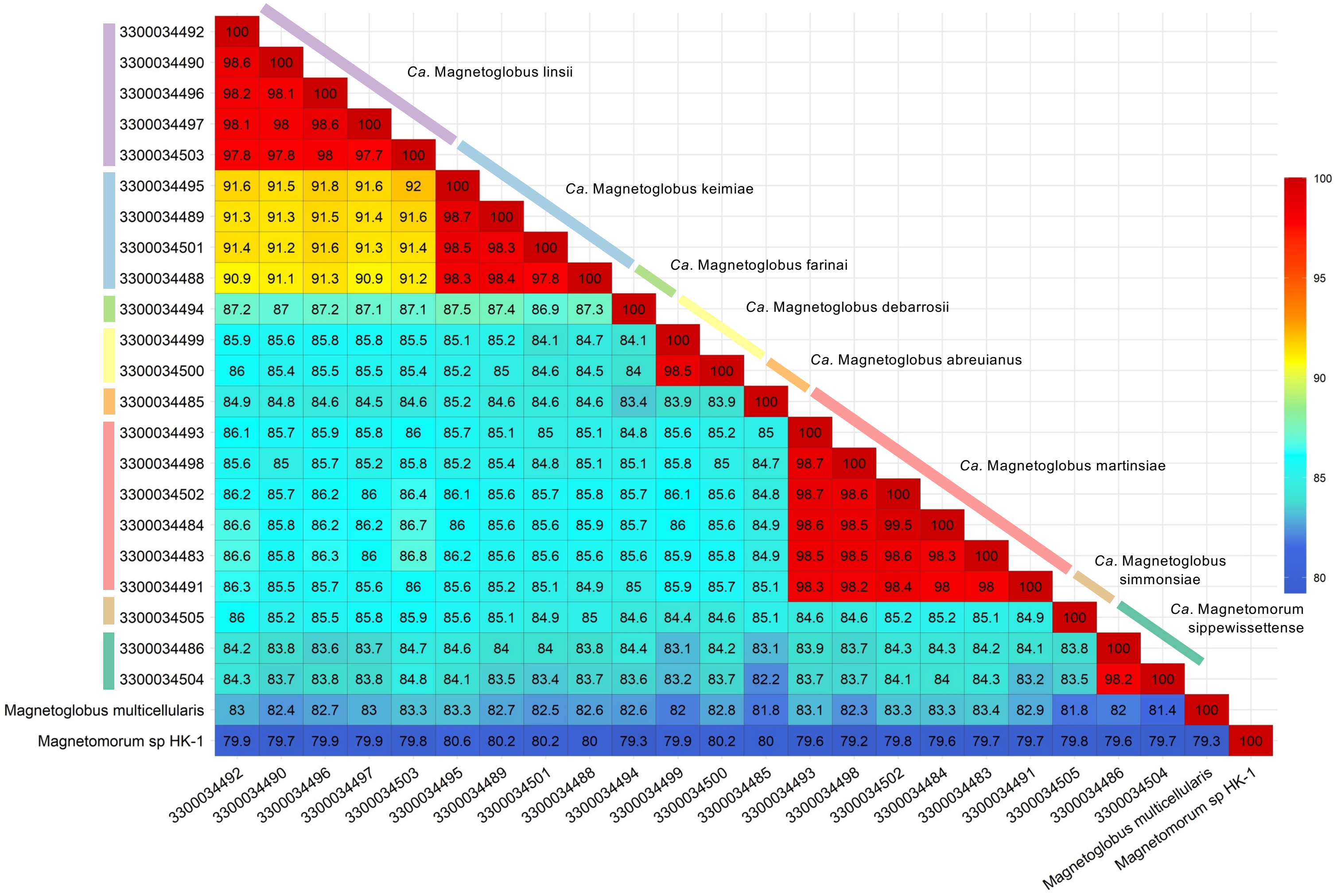

Fig. S5. Genome ANI comparing each of the 22 individual SCMs with the two publicly available reference genomes (*Ca.* M. multicellularis and *Ca.* Magnetomorum sp. HK-1). ANI values are shown within boxes.
