# Supplementary material for: Multicellular magnetotactic bacteria are genetically heterogeneous consortia with metabolically differentiated cells
Source: PLoS Biol. 2024 Jul 11;22(7):e3002638. doi: 10.1371/journal.pbio.3002638 (PMC11239054; doi:10.1371/journal.pbio.3002638)
Supplement: S6 Fig — The analysis suggested that most genes were subject to negative (purifying) selection. On the other hand, genes without functional annotation (“no hits”) were frequently subject to positive selection. The data underlying this figure can be found in Table F in S2 Appendix. (PDF) [file pbio.3002638.s006.pdf]

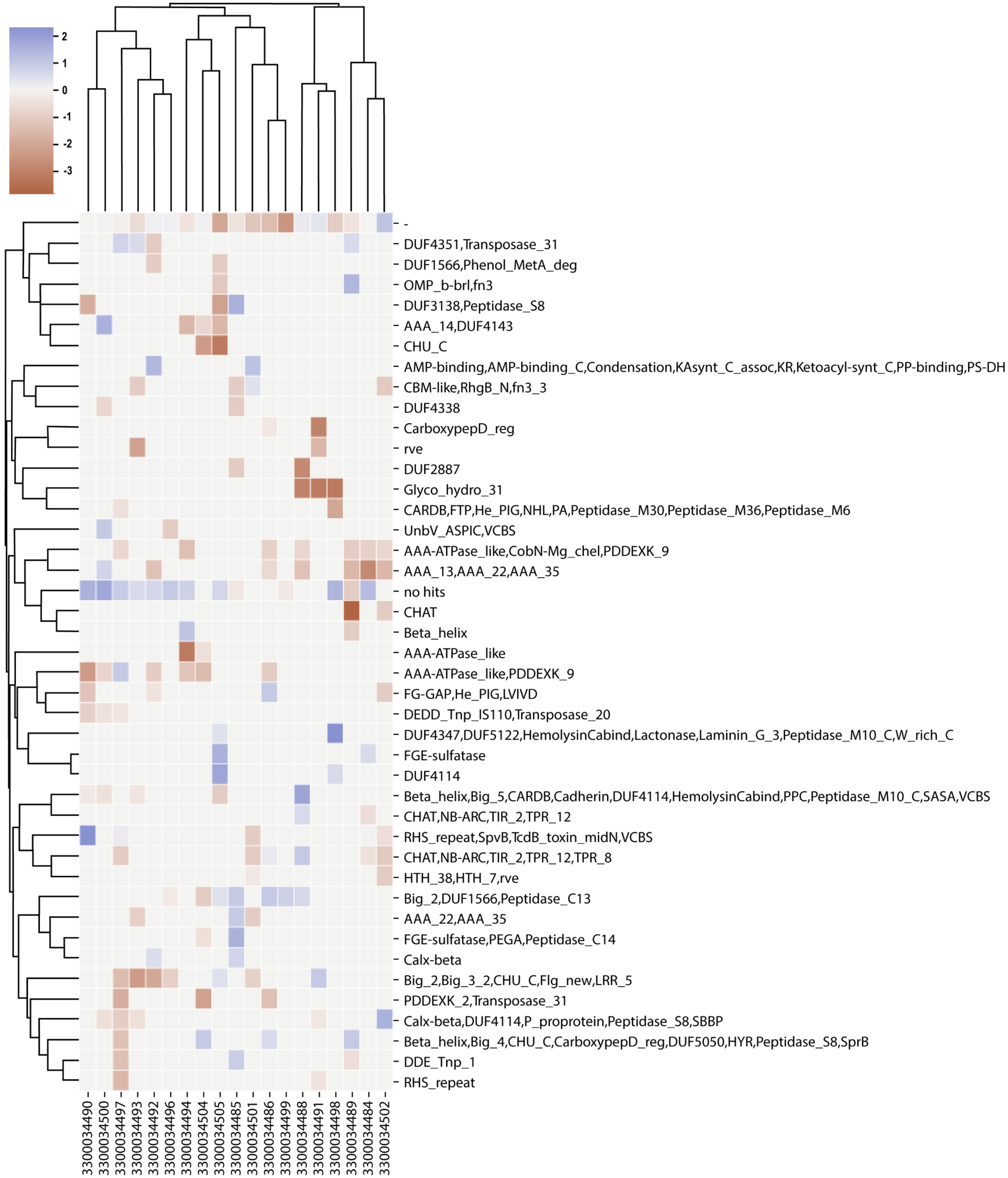

Fig. S6. Heatmap and cluster analysis of pfams annotation of individual SNPs showing the $\log_2$ ratio of non-synonymous to synonymous substitutions (dN/dS) for the SNP differences contained within each SCM. The analysis suggested that most genes were subject to negative (purifying) selection. On the other hand, genes without functional annotation ("no hits") were frequently subject to positive selection. The data underlying this Figure can be found in Table F in S2 Appendix.
