# Supplementary material for: Multicellular magnetotactic bacteria are genetically heterogeneous consortia with metabolically differentiated cells
Source: PLoS Biol. 2024 Jul 11;22(7):e3002638. doi: 10.1371/journal.pbio.3002638 (PMC11239054; doi:10.1371/journal.pbio.3002638)
Supplement: S7 Fig — Vertical lines show peaks corresponding to polyhydroxybutyrate (blue), triglycerides (gold), and exopolysaccharides (pink). Wavenumbers corresponding to peaks are listed in Table F in S2 Appendix. The large peak at approximately 335 cm−1 is assigned to the magnetosome crystal greigite, which has previously been shown for MMB from the same site (Schaible and colleagues). Inset image shows an MMB consortium stained with Nile Red, indicating C-H rich droplets within cells. The contrast and brightness of the image has been increased for better visualization. Scale bar is 5 μm. (PDF) [file pbio.3002638.s007.pdf]

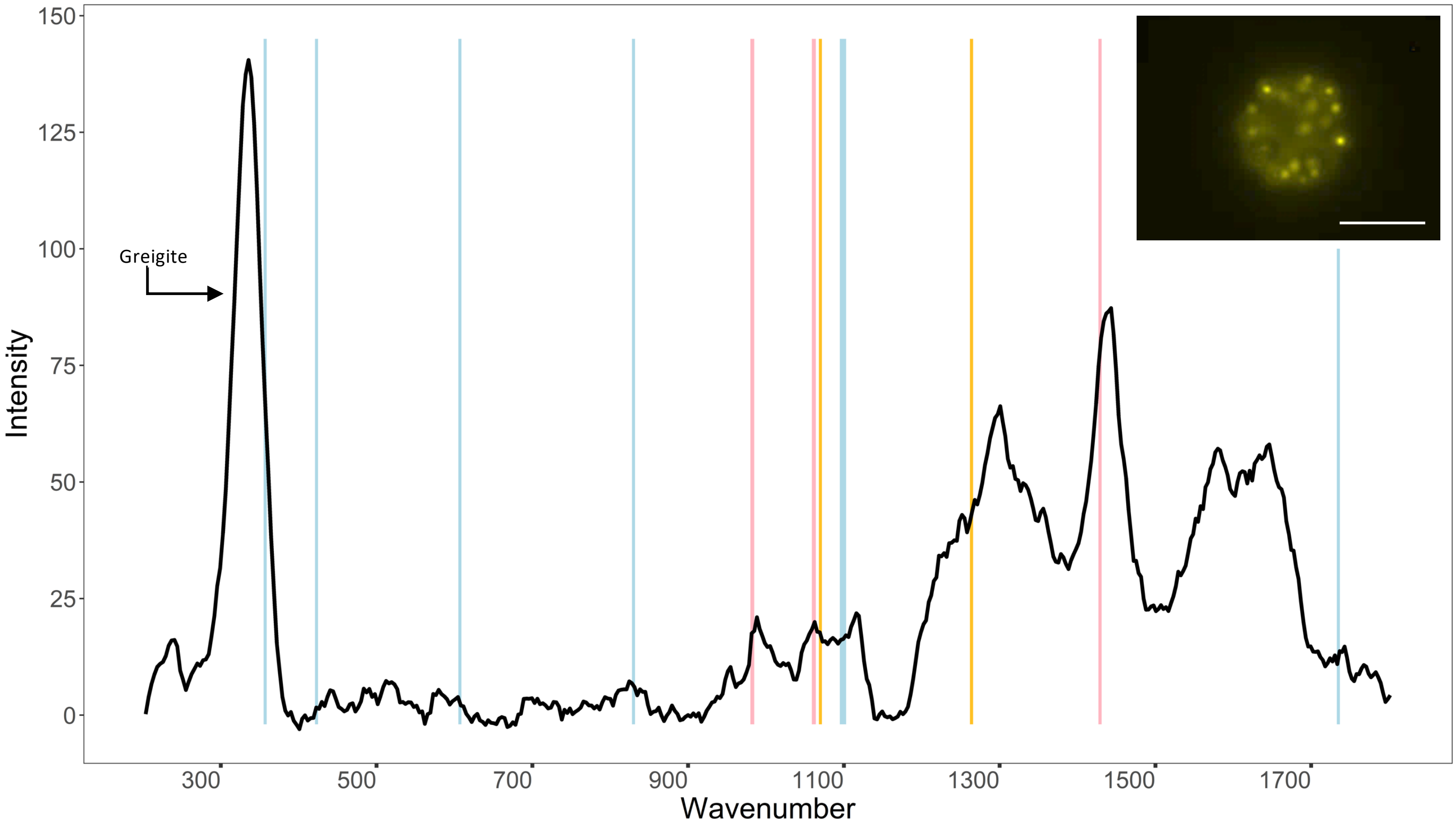

Fig. S7. Representative Raman spectrum of a MMB using a 532 nm laser. Vertical lines show peaks corresponding to polyhydroxybutyrate (blue), triglycerides (gold), and exopolysaccharides (pink). Wavenumbers corresponding to peaks are listed in Table H in S2 Appendix. The large peak at ~335 cm^{-1} is assigned to the magnetosome crystal greigite, which has previously been shown for MMB from the same site (Schaible *et al*., 2022). Inset image shows a MMB consortium stained with Nile Red, indicating C-H rich droplets within cells. The contrast and brightness of the image has been increased for better visualization. Scale bar is 5 μm.
