# Supplementary material for: Multicellular magnetotactic bacteria are genetically heterogeneous consortia with metabolically differentiated cells
Source: PLoS Biol. 2024 Jul 11;22(7):e3002638. doi: 10.1371/journal.pbio.3002638 (PMC11239054; doi:10.1371/journal.pbio.3002638)
Supplement: S8 Fig — (A1-3) Control sample of MMB incubated without cellulase. (B1-3) After treatment with cellulase the surface of MMB consortia was noticeably eroded as compared to the control. Both samples were incubated for 1 h under otherwise identical conditions (pH, temperature, and osmolarity). All scale bars are 1 μm. (PDF) [file pbio.3002638.s008.pdf]

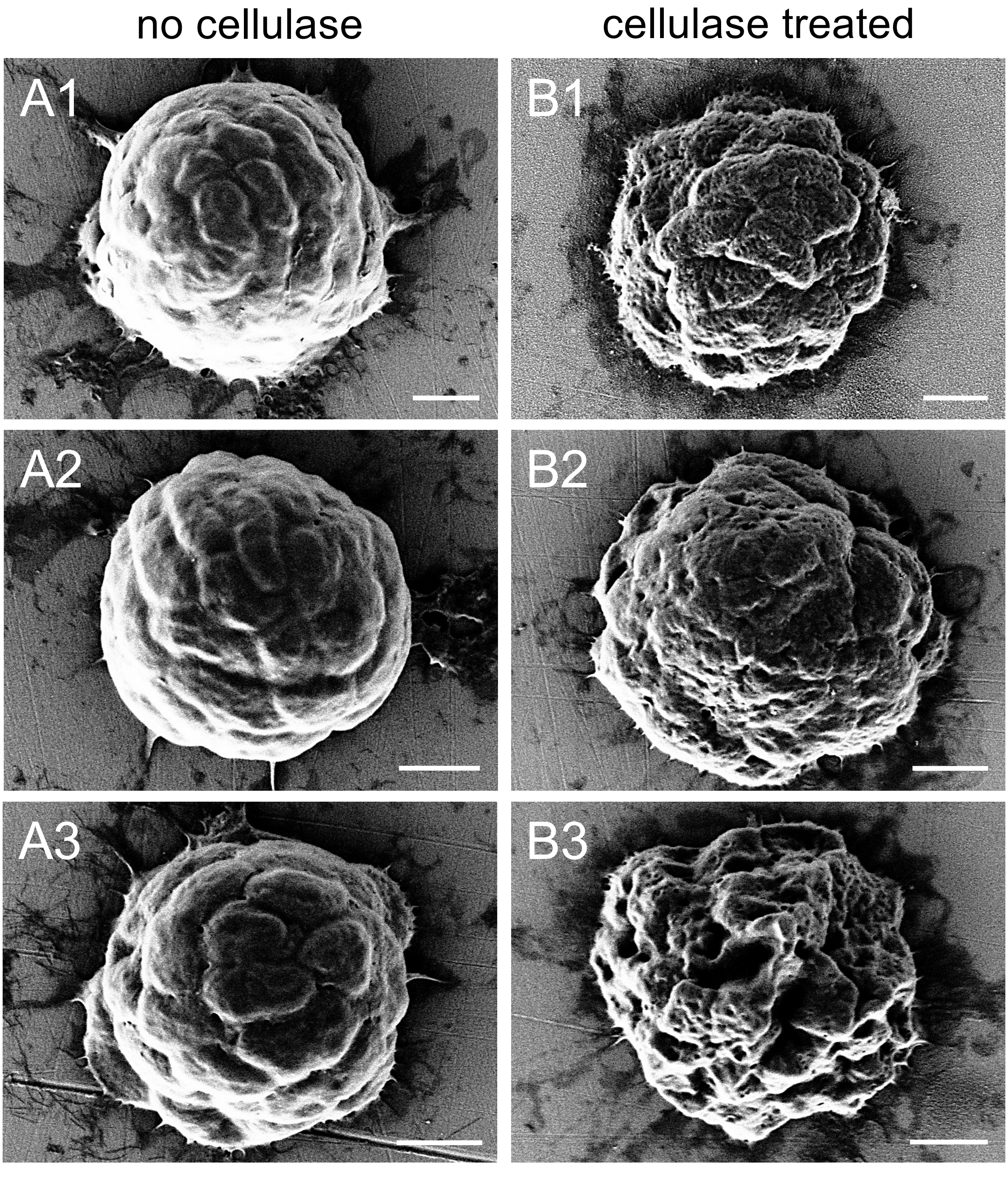

Fig. S8. Cellulase treatment of MMB. (**A1-3**) Control sample of MMB incubated without cellulase. (**B1-3**) After treatment with cellulase the surface of MMB consortia was noticeably eroded as compared to the control. Both samples were incubated for 1 hr under otherwise identical conditions (pH, temperature, and osmolarity). All scale bars are 1 μm.
