# Supplementary material for: Multicellular magnetotactic bacteria are genetically heterogeneous consortia with metabolically differentiated cells
Source: PLoS Biol. 2024 Jul 11;22(7):e3002638. doi: 10.1371/journal.pbio.3002638 (PMC11239054; doi:10.1371/journal.pbio.3002638)
Supplement: S9 Fig — (A) Image of the 15 cm core taken from the West end of sampling site prior to being sectioned into 1 cm horizons from which MMB were enriched for quantification by FISH. (B) DOPE-FISH analysis of MMB Groups 2 (red) and 5 (green) shown in panel (B) and Groups 1 (green), 3 (yellow), and 4 (red) shown in panel (C). MMB not detected by the respective FISH probes are shown in the blue DAPI counterstain in the microscopy images. Scale bars are 5 μm. Bar plots show the abundance of each MMB group as determined by DOPE-FISH for each centimeter of the sediment core shown in panel (A). Unlabeled populations are MMB that were stained with DAPI but were not detected by the FISH probes used in the 2 separate experiments and are shown in gray. Consistent with results from SCM and previous 16S rRNA gene abundance studies (Simmons and Edwards, 2007) in LSSM, Group 1 numerically dominate the MMB population. FISH probes used in this experiment are detailed in Table I in S2 Appendix. Statistical analyses were performed using a pairwise t test with the Bonferroni p-adjusted method. The data underlying this figure can be found in Table P in S2 Appendix. Photo by George Schaible. (PDF) [file pbio.3002638.s009.pdf]

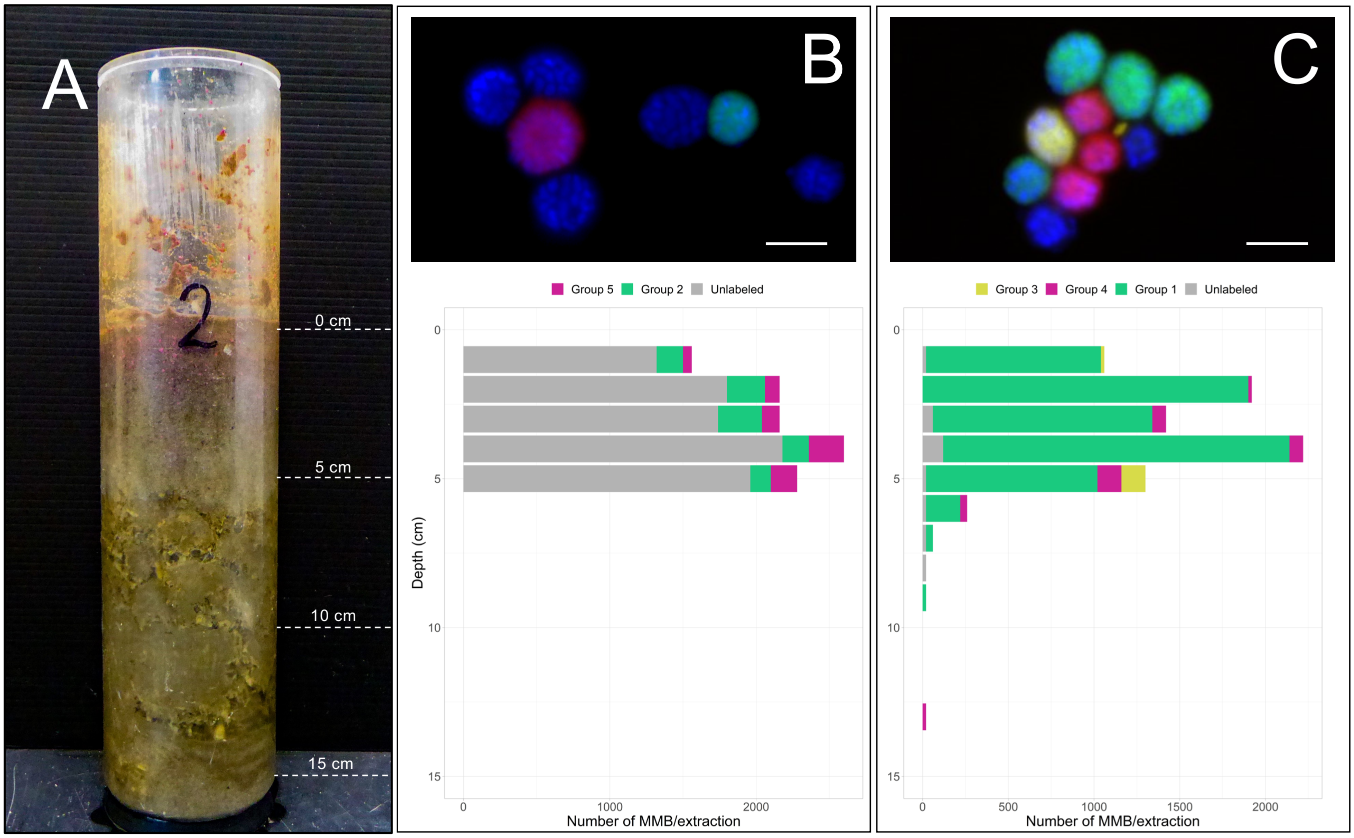

Fig. S9. Fractional abundance of MMB groups by depth in LSSM. (**A**) Image of the 15 cm core taken from the West end of sampling site prior to being sectioned into 1 cm horizons from which MMB were enriched for quantification by FISH. (**B**) DOPE-FISH analysis of MMB Groups 2 (red) and 5 (green) shown in panel (**B**) and Groups 1 (green), 3 (yellow) and 4 (red) shown in panel (**C**). MMB not detected by the respective FISH probes are shown in the blue DAPI counterstain in the microscopy images. Scale bars are 5 µm. Bar plots show the abundance of each MMB group as determined by DOPE-FISH for each centimeter of the sediment core shown in panel **A**. Unlabeled populations are MMB that were stained with DAPI but were not detected by the FISH probes used in the two separate experiments and are shown in gray. Consistent with results from SCM and previous 16S rRNA gene abundance studies (Simmons and Edwards 2007) in LSSM, Group 1 numerically dominate the MMB population. FISH probes used in this experiment are detailed in SI Appendix Table S9. Statistical analyses were performed using a pairwise t-test with the Bonferroni p-adjusted method. The data underlying this Figure can be found in Table P in S2 Appendix. Photo by George Schaible.
