# Supplementary material for: Multicellular magnetotactic bacteria are genetically heterogeneous consortia with metabolically differentiated cells
Source: PLoS Biol. 2024 Jul 11;22(7):e3002638. doi: 10.1371/journal.pbio.3002638 (PMC11239054; doi:10.1371/journal.pbio.3002638)
Supplement: S10 Fig — (A) 1 cm sediment core horizons were incubated in the presence of the methionine analogue HPG and magnetically enriched MMB stained via azide-alkyne click chemistry with Alexa Fluor 405 to show relative activity of Group 1 MMB as a factor of depth in the sediment. The vertical line within each box shows the median and the whisker shows the range of the data. Dots represent individual MMB that were measured and analyzed using the software package Daime. Data points that were more than 2 standard deviations of the mean are shown as individual points past the whicker. The analysis showed that there is a statistically relevant difference in the activity of MMB from 1 cm depth to 2 to 3 cm and again from 2 to 3 cm to the 4 to 5 cm depth. (B) Exemplary epifluorescence microscopy image of click-stained MMB. (C) Overlay epifluorescence microscopy image of FISH-labeled MMB shown in panel B. Group 1 is shown in green, Group 3 in yellow, and Group 4 in red. All scale bars are 5 μm. All statistically differences are shown: ** = p < 3.9 × 10^–3^, *** = p < 3.5 × 10^–4^. FISH probes used in this experiment are detailed in Table I in S2 Appendix. Statistical analyses were performed using a pairwise t test with the Bonferroni p-adjusted method. The data underlying this figure can be found in Table Q in S2 Appendix. (PDF) [file pbio.3002638.s010.pdf]

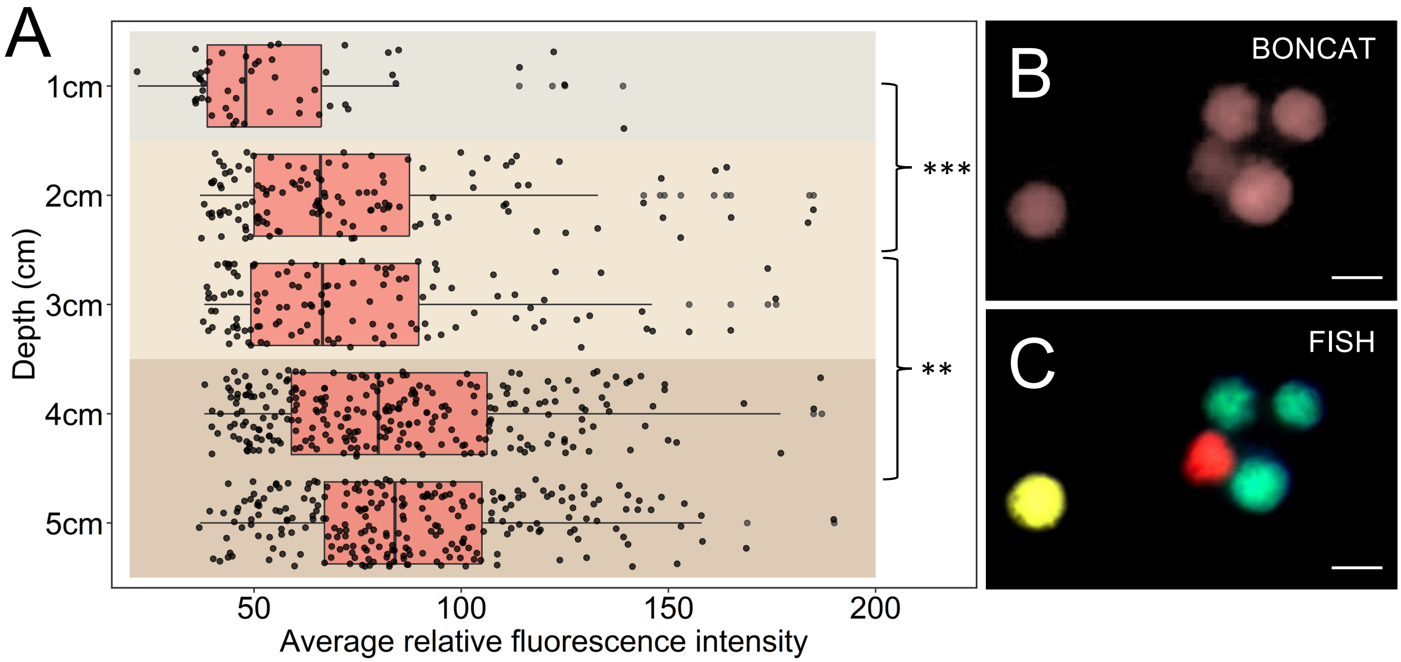

Fig. S10. Anabolic activity of MMB inhabiting the top 6 cm of LSSM sediment as measured by BONCAT. (**A**) 1 cm sediment core horizons were incubated in the presence of the methionine analogue HPG and magnetically enriched MMB stained via azide-alkyne click chemistry with Alexa Fluor 405 to show relative activity of Group 1 MMB as a factor of depth in the sediment. The vertical line within each box shows the median and the whisker shows the range of the data. Dots represent individual MMB that were measured and analyzed using the software package Daime. Data points that were more than two standard deviations of the mean are shown as individual points past the whicker. The analysis showed that there is a statistically relevant difference in the activity of MMB from 1 cm depth to 2-3 cm and again from 2-3 cm to the 4-5 cm depth. (**B**) Exemplary epifluorescence microscopy image of click-stained MMB. (**C**) Overlay epifluorescence microscopy image of FISH-labeled MMB shown in panel **B**. Group 1 is shown in green, Group 3 in yellow, and Group 4 in red. All scale bars are 5 µm. All statistically differences are shown: ** = $p < 3.9 \times 10^{-3}$, *** = $p < 3.5 \times 10^{-4}$. FISH probes used in this experiment are detailed in SI Appendix Table S9. Statistical analyses were performed using a pairwise t-test with the Bonferroni p-adjusted method. The data underlying this Figure can be found in SI Appendix Table S17.
