# Supplementary material for: Multicellular magnetotactic bacteria are genetically heterogeneous consortia with metabolically differentiated cells
Source: PLoS Biol. 2024 Jul 11;22(7):e3002638. doi: 10.1371/journal.pbio.3002638 (PMC11239054; doi:10.1371/journal.pbio.3002638)
Supplement: S11 Fig — The analysis shows that MMB in Group 1 anabolize acetate at a statistically greater rate than Groups 3 and 4 (p < 8.9 × 10^−3^). Group 1 also incorporated more bicarbonate than Group 4 (p < 2.4 × 10^−2^), although Group 4 only contained 4 samples to compare. Statistical analyses were performed using a pairwise t test with the Bonferroni p-adjusted method. The data underlying this figure can be found in Table K in S2 Appendix. (PDF) [file pbio.3002638.s011.pdf]

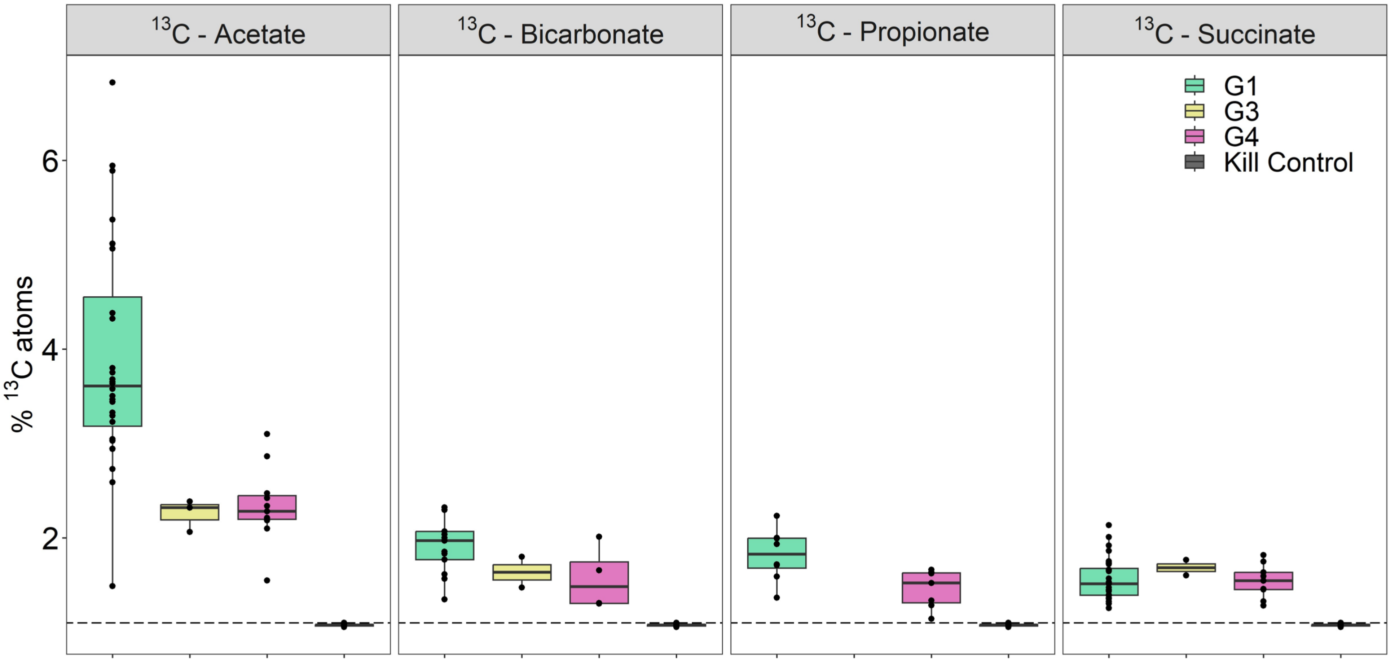

Fig. S11. Comparison of ^{13}C-labeled substrate incorporation by MMB Groups 1, 3, and 4 using NanoSIMS analysis of mass ratio $^{13}C^{12}C/^{12}C_2$. The analysis shows that MMB in Group 1 anabolize acetate at a statistically greater rate than Groups 3 and 4 ($p < 8.9 \times 10^{-3}$). Group 1 also incorporated more bicarbonate than Group 4 ($p < 2.4 \times 10^{-2}$), although Group 4 only contained four samples to compare. Statistical analyses were performed using a pairwise t-test with the Bonferroni p-adjusted method. The data underlying this Figure can be found in Table K in S2 Appendix.
