# Supplementary material for: Multicellular magnetotactic bacteria are genetically heterogeneous consortia with metabolically differentiated cells
Source: PLoS Biol. 2024 Jul 11;22(7):e3002638. doi: 10.1371/journal.pbio.3002638 (PMC11239054; doi:10.1371/journal.pbio.3002638)
Supplement: S12 Fig — The (A) taxonomy (DOPE-FISH), (B) morphology (SEM), (C) distribution of sulfur (NanoSIMS, mass 32; a proxy for the presence of sulfur-containing magnetosomes), and (D) uptake of 1,2-13C2-labeled acetate (NanoSIMS, HSI image showing mass ratio 13C12C/12C2). Scale bars are 5 μm. The HSI mass ratio color scale in (D) is 1.1%–5% atom percent. (PDF) [file pbio.3002638.s012.pdf]

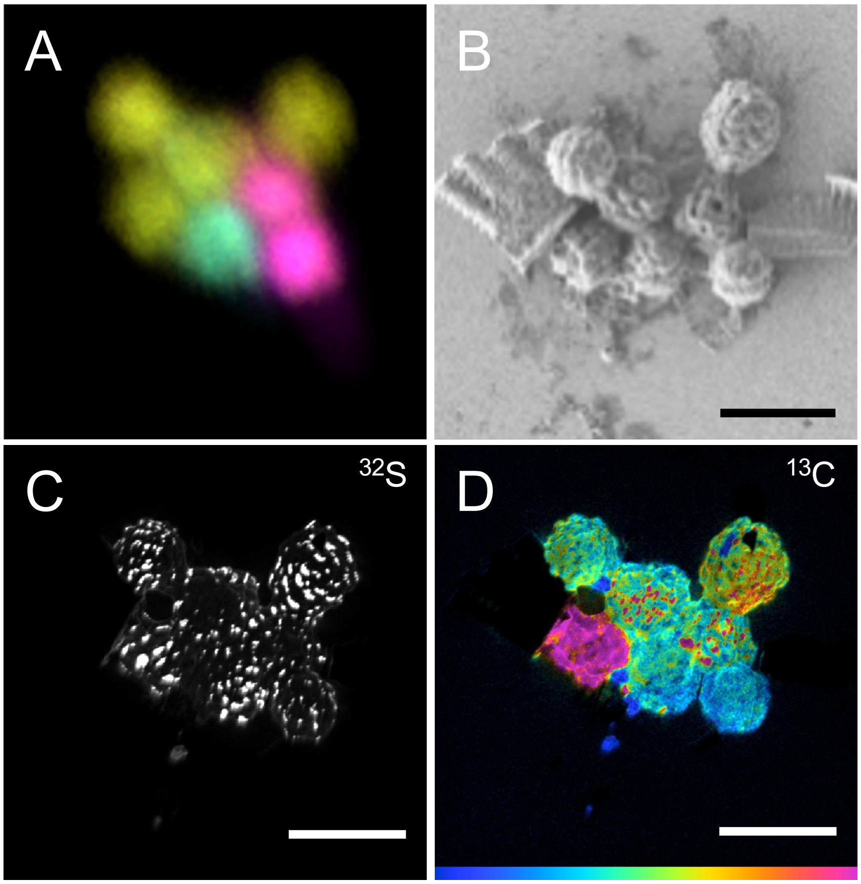

Fig. S12. Correlative imaging of MMB. The (**A**) taxonomy (DOPE-FISH), (**B**) morphology (SEM), (**C**) distribution of sulfur (NanoSIMS, mass 32; a proxy for the presence of sulfur-containing magnetosomes), and (**D**) uptake of 1,2-$^{13}C_2$-labeled acetate (NanoSIMS, HSI image showing mass ratio $^{13}C^{12}C/^{12}C_2$). Scale bars are 5 µm. The HSI mass ratio color scale in *D* is 1.1% - 5% atom percent.
