# Supplementary material for: Multicellular magnetotactic bacteria are genetically heterogeneous consortia with metabolically differentiated cells
Source: PLoS Biol. 2024 Jul 11;22(7):e3002638. doi: 10.1371/journal.pbio.3002638 (PMC11239054; doi:10.1371/journal.pbio.3002638)
Supplement: S13 Fig — Because the in situ incubation incurred particles that were not of interest (e.g., diatoms and particulates), the ROIs were hand drawn around each MMB using the mass 26.00 (12C14N) channel to avoid incorporation of exogenous material in the analysis. (A1) 13C-acetate, (A2) 12C-acetate, (B1) 13C-bicarbonate, (B2) 12C-bicarbonate, (C1) 13C-propionate, (C2) 12C-propionate, (D1) 13C- succinate, (D2) 12C-succinate, (E) 13C-acetate kill control, (F) negative control. ROIs are shown in green and red outlines. (PDF) [file pbio.3002638.s013.pdf]

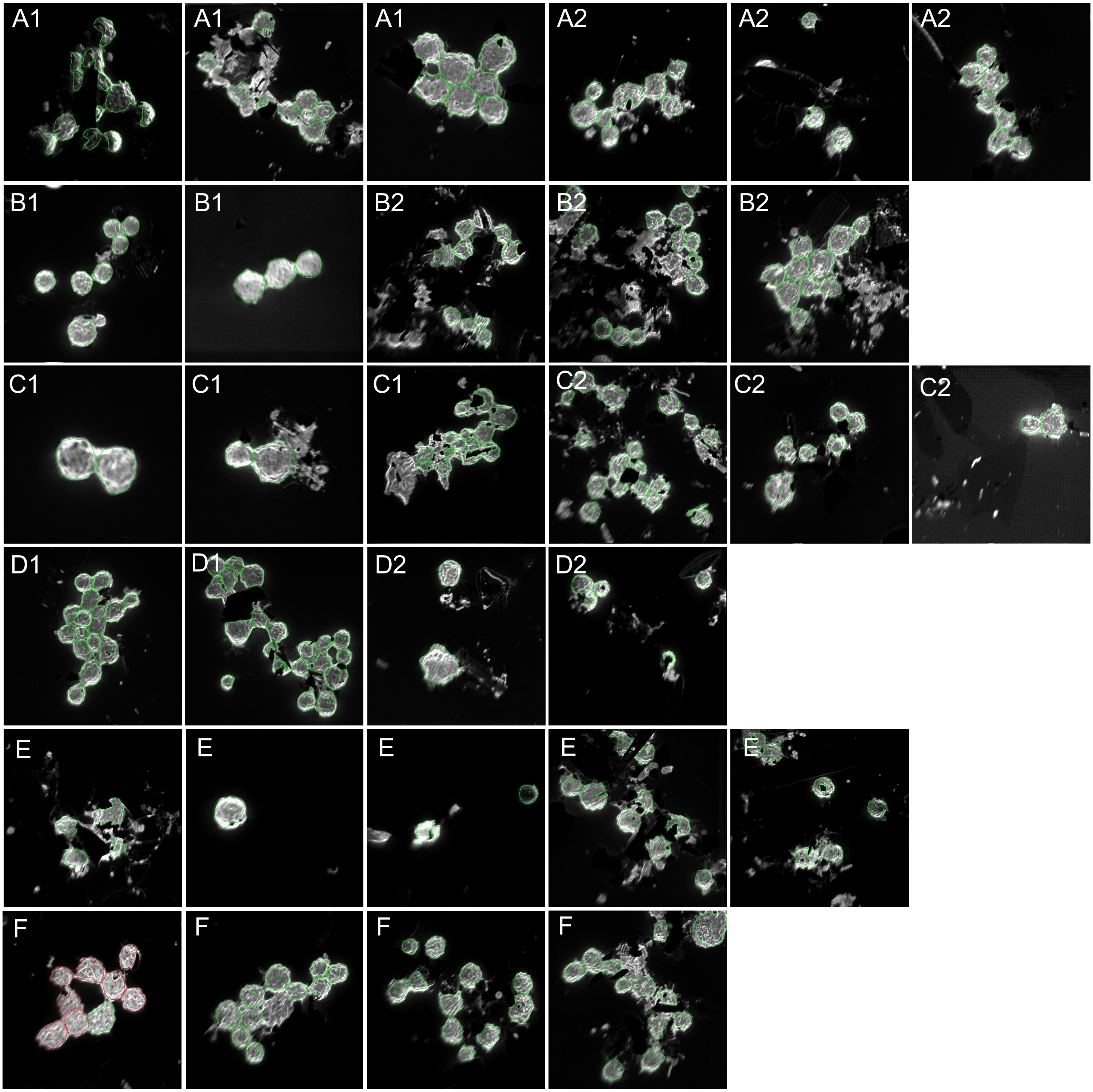

Fig. S13. ROIs for NanoSIMS substrate analysis shown in Fig. 5 of main text. Because the *in situ* incubation incurred particles that were not of interest (*e.g.*, diatoms and particulates), the ROIs were hand drawn around each MMB using the mass 26.00 ($^{12}C^{14}N$) channel as to avoid incorporation of exogenous material in the analysis. (**A1**) ^{13}C-acetate, (**A2**) ^{12}C-acetate, (**B1**) ^{13}C-bicarbonate, (**B2**) ^{12}C-bicarbonate, (**C1**) ^{13}C-propionate, (**C2**) ^{12}C-propionate, (**D1**) ^{13}C-succinate, (**D2**) ^{12}C-succinate, (**E**) ^{13}C-acetate kill control, (**F**) negative control. ROIs are shown in green and red outlines.
