# Supplementary material for: Multicellular magnetotactic bacteria are genetically heterogeneous consortia with metabolically differentiated cells
Source: PLoS Biol. 2024 Jul 11;22(7):e3002638. doi: 10.1371/journal.pbio.3002638 (PMC11239054; doi:10.1371/journal.pbio.3002638)
Supplement: S14 Fig — As to avoid introducing bias into the selection of hotspot ROIs, thresholding in ImageJ was used to automatically select for ROIs, as outlined in the methods. The respective mass image was used for hotspot thresholding and ROI selection. ROIs for whole consortia were hand drawn. All ROIs are show in red outlines. (PDF) [file pbio.3002638.s014.pdf]

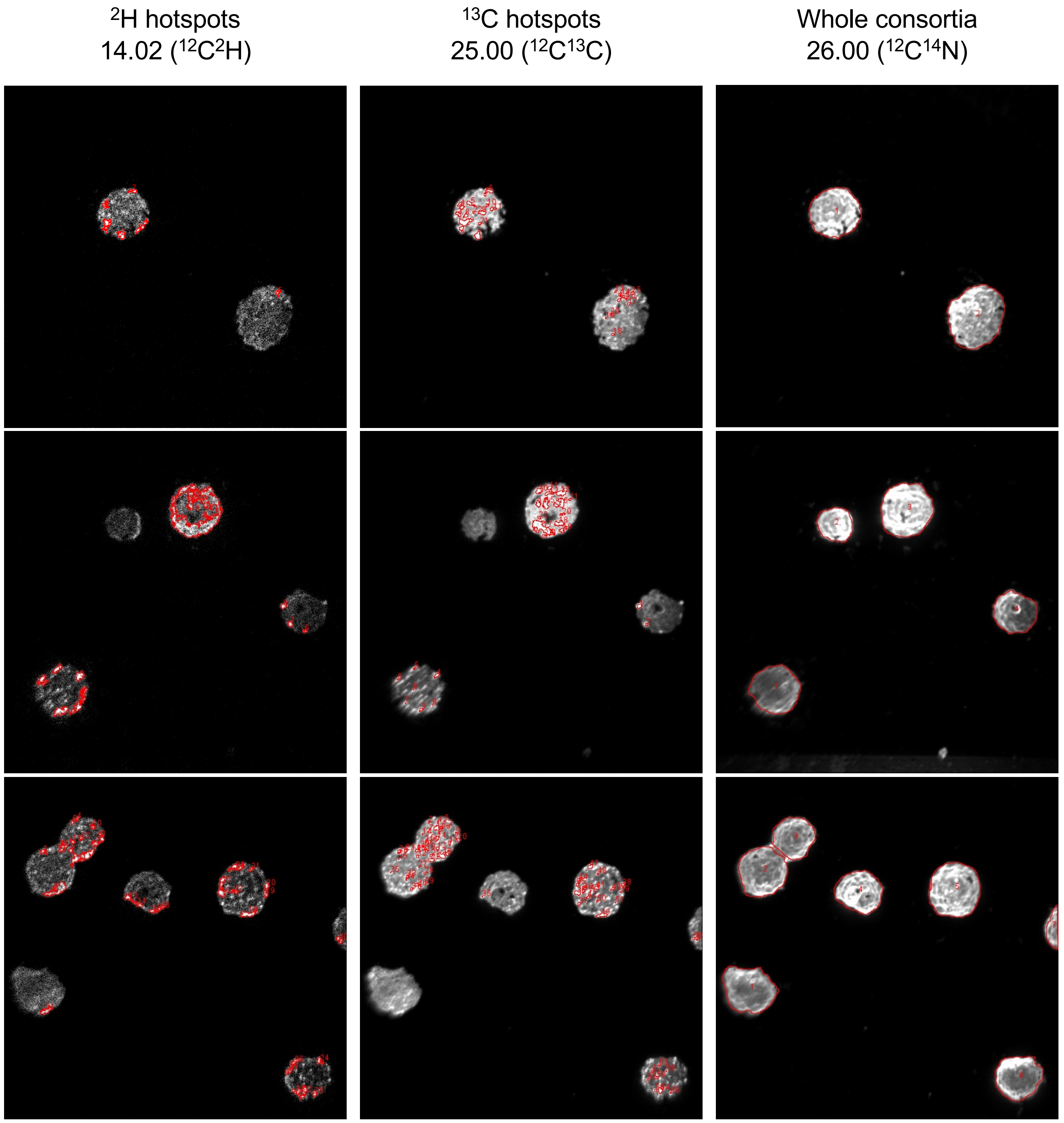

Fig. S14. ROIs for NanoSIMS hotspot analysis shown in Fig. 6 of main text. As to avoid introducing bias into the selection of hotspot ROIs, thresholding in ImageJ was used to automatically select for ROIs, as outlined in the methods. The respective mass image was used for hotspot thresholding and ROI selection. ROIs for whole consortia were hand drawn. All ROIs are show in red outlines.
