# Supplementary material for: Multicellular magnetotactic bacteria are genetically heterogeneous consortia with metabolically differentiated cells
Source: PLoS Biol. 2024 Jul 11;22(7):e3002638. doi: 10.1371/journal.pbio.3002638 (PMC11239054; doi:10.1371/journal.pbio.3002638)
Supplement: S15 Fig — Median filter ratio radius effect on HSI NanoSIMS images of 13C and 2H hotspots (A–C) Mass ratio (2H12C/1H12C) of MMB labeled with deuterium oxide (2H2O). (D–F) Mass ratio (13C12C/12C2) of the same MMB shown in (A–C) but labeled with 1,2-13C2-labeled acetate. For these images, the median filter ratio radius was increased to show the effect of noise reduction and localization of isotope label within consortia. A higher filter radius reveals isolated areas of the respective isotope label within MMB, though for a radius >5, the label is averaged over an area greater than the size of a single cell within the consortium, thus losing cellular resolution. Independent of the radius chosen, hot spots remain visible. (PDF) [file pbio.3002638.s015.pdf]

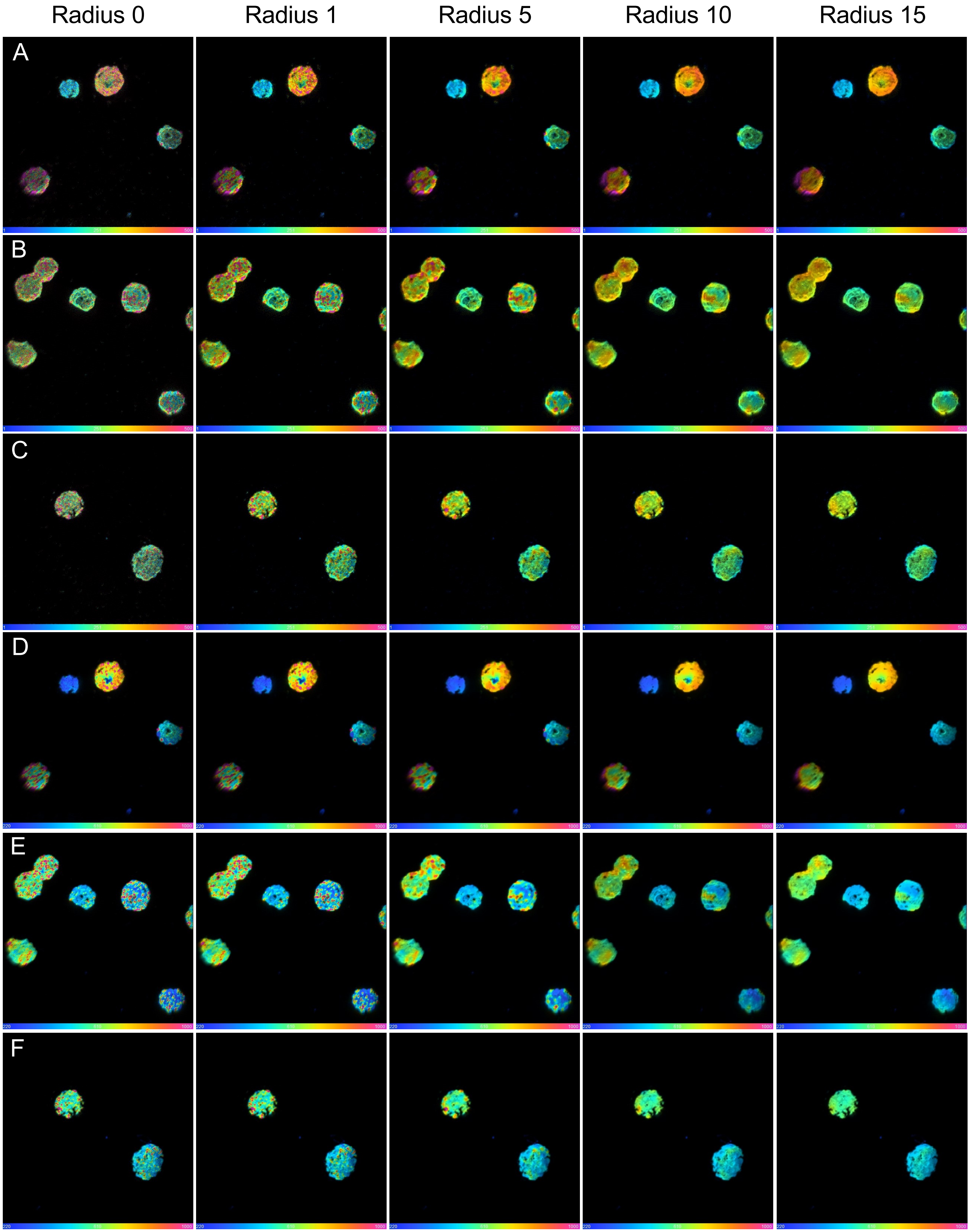

Fig. S15. Median filter ratio radius effect on HSI NanoSIMS images of ^{13}C and ^{2}H hotspots. (**A-C**) Mass ratio ($^{2}H^{12}C/^{1}H^{12}C$) of MMB labeled with deuterium oxide ($^{2}H_2O$). (**D-F**) Mass ratio ($^{13}C^{12}C/^{12}C_2$) of the same MMB shown in **A-C** but labeled with 1,2-$^{13}C_2$-labeled acetate. For these images, the median filter ratio radius was increased to show the effect of noise reduction and localization of isotope label within consortia. A higher filter radius reveals isolated areas of the respective isotope label within MMB, though for a radius > 5, the label is averaged over an area greater than the size of a single cell within the consortium, thus losing cellular resolution. Independent of the radius chosen, hot spots remain visible.
