# Supplementary material for: Multicellular magnetotactic bacteria are genetically heterogeneous consortia with metabolically differentiated cells
Source: PLoS Biol. 2024 Jul 11;22(7):e3002638. doi: 10.1371/journal.pbio.3002638 (PMC11239054; doi:10.1371/journal.pbio.3002638)
Supplement: S16 Fig — (A) Gray-scale images of individual MMB stained via azide-alkyne click chemistry with Alexa Fluor 488. (B) The same consortia shown in (A) that have been rotationally averaged in Eman2 software. The relative fluorescence intensity was standardized for all samples prior to analysis. (PDF) [file pbio.3002638.s016.pdf]

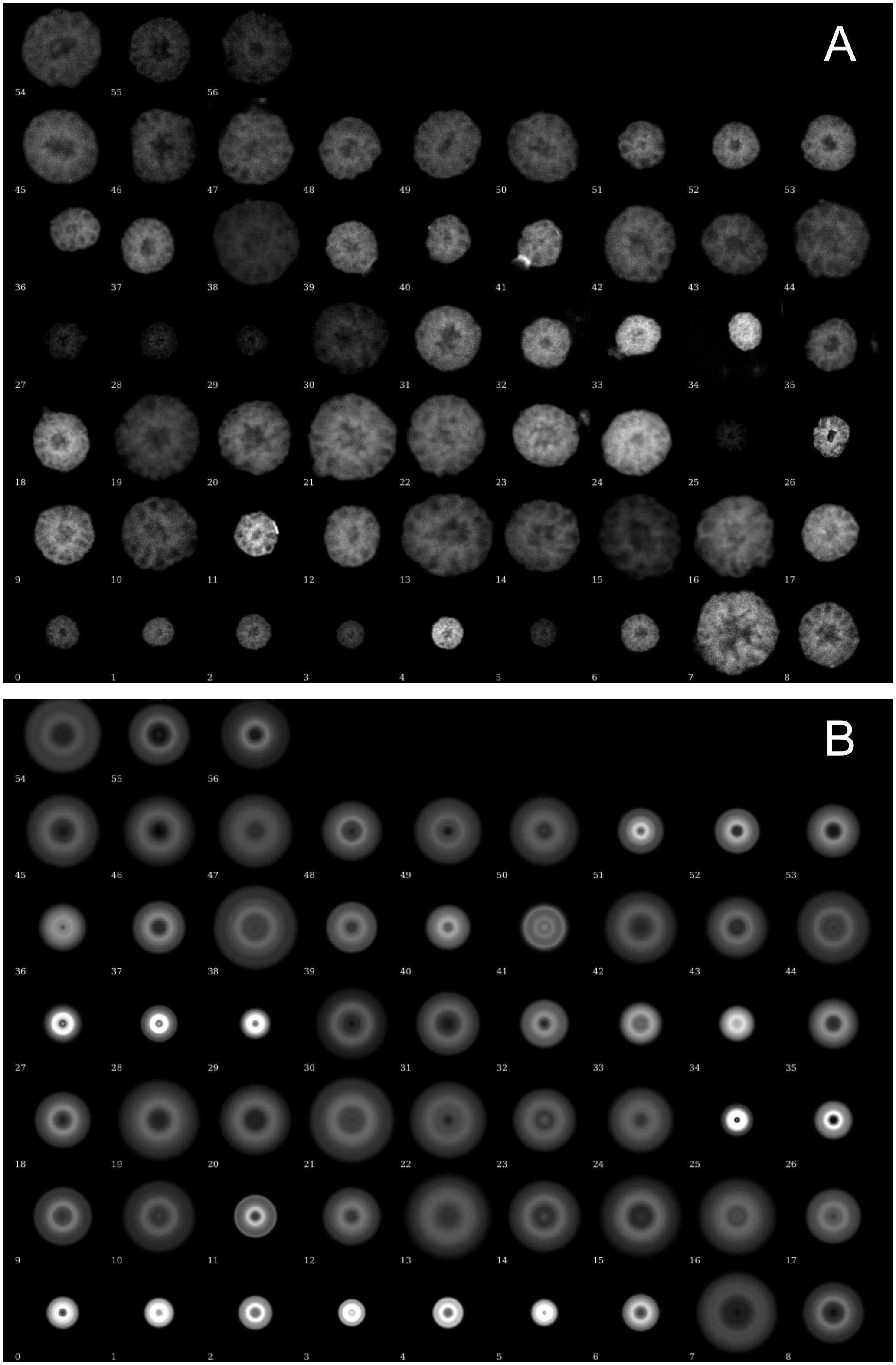

Fig. S16. Anabolic activity within individual consortia. (**A**) Gray-scale images of individual MMB stained via azide-alkyne click chemistry with Alexa Fluor 488. (**B**) The same consortia shown in **A** that have been rotationally averaged in Eman2 software. The relative fluorescence intensity was standardized for all samples prior to analysis.
