# Supplementary material for: Multicellular magnetotactic bacteria are genetically heterogeneous consortia with metabolically differentiated cells
Source: PLoS Biol. 2024 Jul 11;22(7):e3002638. doi: 10.1371/journal.pbio.3002638 (PMC11239054; doi:10.1371/journal.pbio.3002638)
Supplement: S17 Fig — (A) AHA positive BONCAT E. coli control. (B) AHA negative BONCAT E. coli control. (C) AHA positive BONCAT of MMB. (D) AHA negative BONCAT of MMB. (E) The exposure time was adjusted to allow for visualization of the AHA negative MMB from panel (D), which resulted in a dramatic over exposure (F) of the AHA positive MMB from panel (C). (PDF) [file pbio.3002638.s017.pdf]

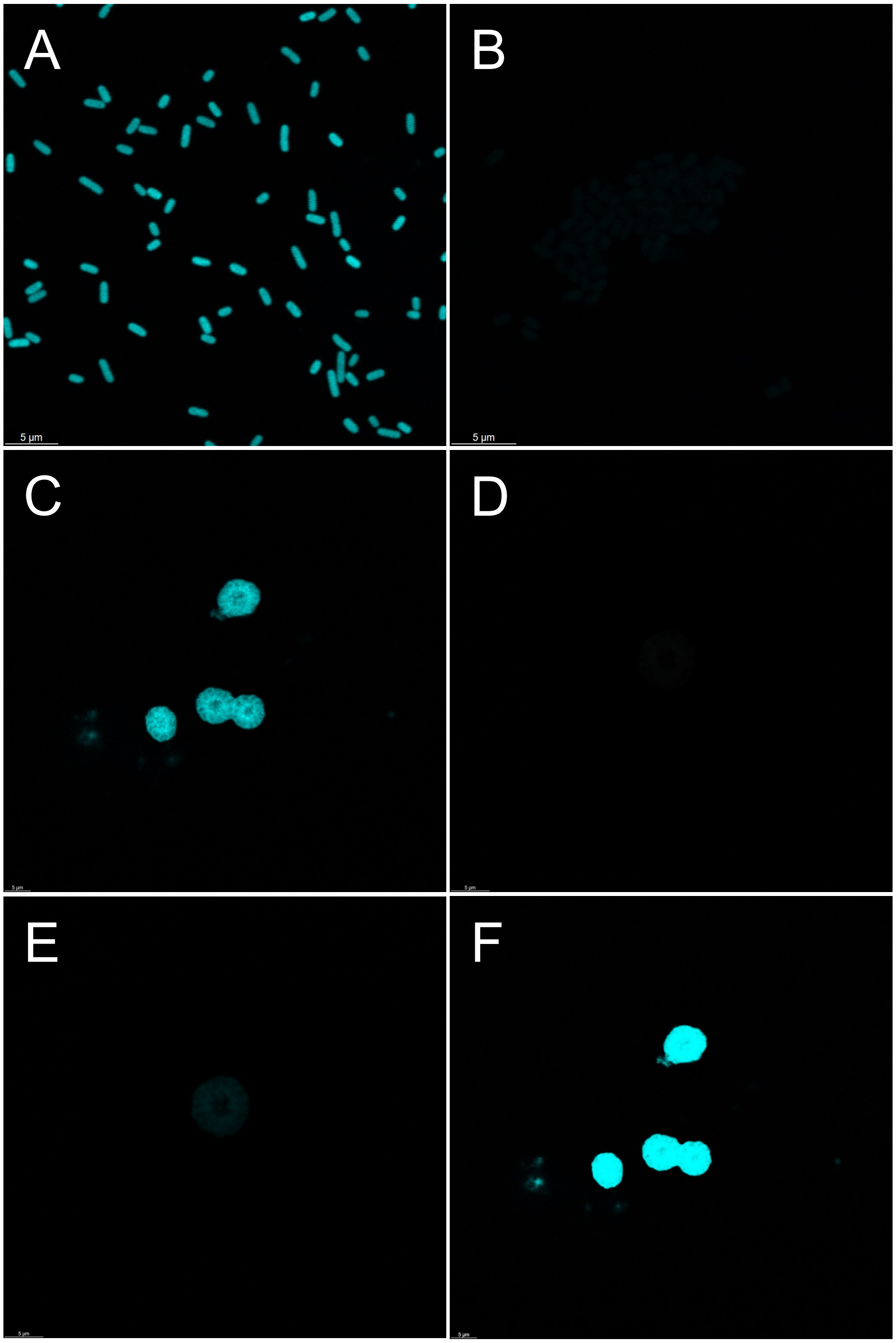


**Fig. S17.** Controls for metabolic differentiation as studied by BONCAT. (**A**) AHA positive BONCAT *E. coli* control. (**B**) AHA negative BONCAT *E. coli* control. (**C**) AHA positive BONCAT of MMB. (**D**) AHA negative BONCAT of MMB. (**E**) The exposure time was adjusted to allow for visualization of the AHA negative MMB from panel **D**, which resulted in a dramatic over exposure (**F**) of the AHA positive MMB from panel **C**.
