# Supplementary material for: Multicellular magnetotactic bacteria are genetically heterogeneous consortia with metabolically differentiated cells
Source: PLoS Biol. 2024 Jul 11;22(7):e3002638. doi: 10.1371/journal.pbio.3002638 (PMC11239054; doi:10.1371/journal.pbio.3002638)
Supplement: S18 Fig — A sorting gate, presumed to contain MMB consortia, was set around particles with a strong 488 nm signal and high side scatter (SSC), indicating a large cell size. Other particles likely are single-cell magnetotactic bacteria or non-magnetotactic bacteria present in the pond water. MMB consortia were sorted into individual wells of a microtiter well plate and 22 MMB consortia were genome sequenced. (PDF) [file pbio.3002638.s018.pdf]

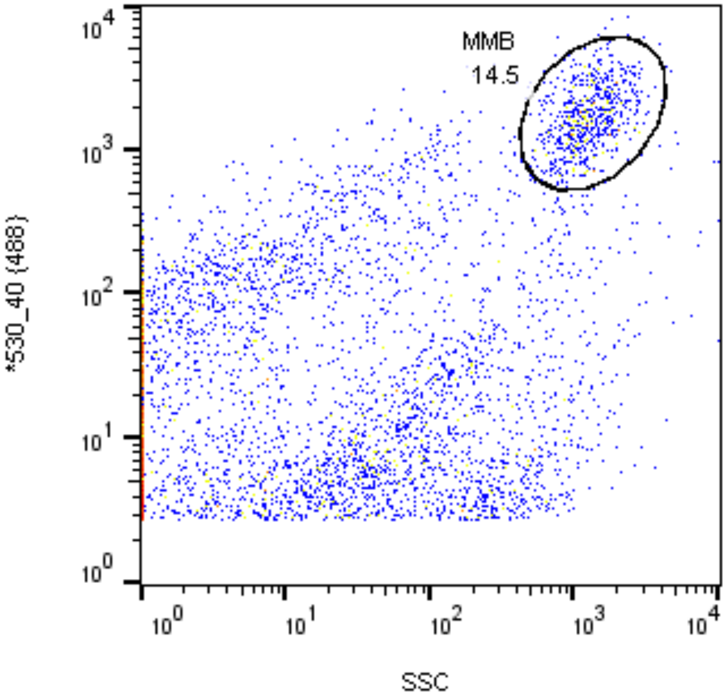

Fig. S18. Fluorescence activated cell sorting of a magnetically enriched sample from tidal pond sediment stained with SYBR Green. A sorting gate, presumed to contain MMB consortia, was set around particles with a strong 488 nm signal and high side scatter (SSC), indicating a large cell size. Other particles likely are single cell magnetotactic bacteria or non-magnetotactic bacteria present in the pond water. MMB consortia were sorted into individual wells of a microtiter well plate and 22 MMB consortia were genome sequenced.
