# Supplementary material for: Multicellular magnetotactic bacteria are genetically heterogeneous consortia with metabolically differentiated cells
Source: PLoS Biol. 2024 Jul 11;22(7):e3002638. doi: 10.1371/journal.pbio.3002638 (PMC11239054; doi:10.1371/journal.pbio.3002638)
Supplement: S19 Fig — The corresponding annotations of colored genes are shown in the legend to the right. (PDF) [file pbio.3002638.s019.pdf]

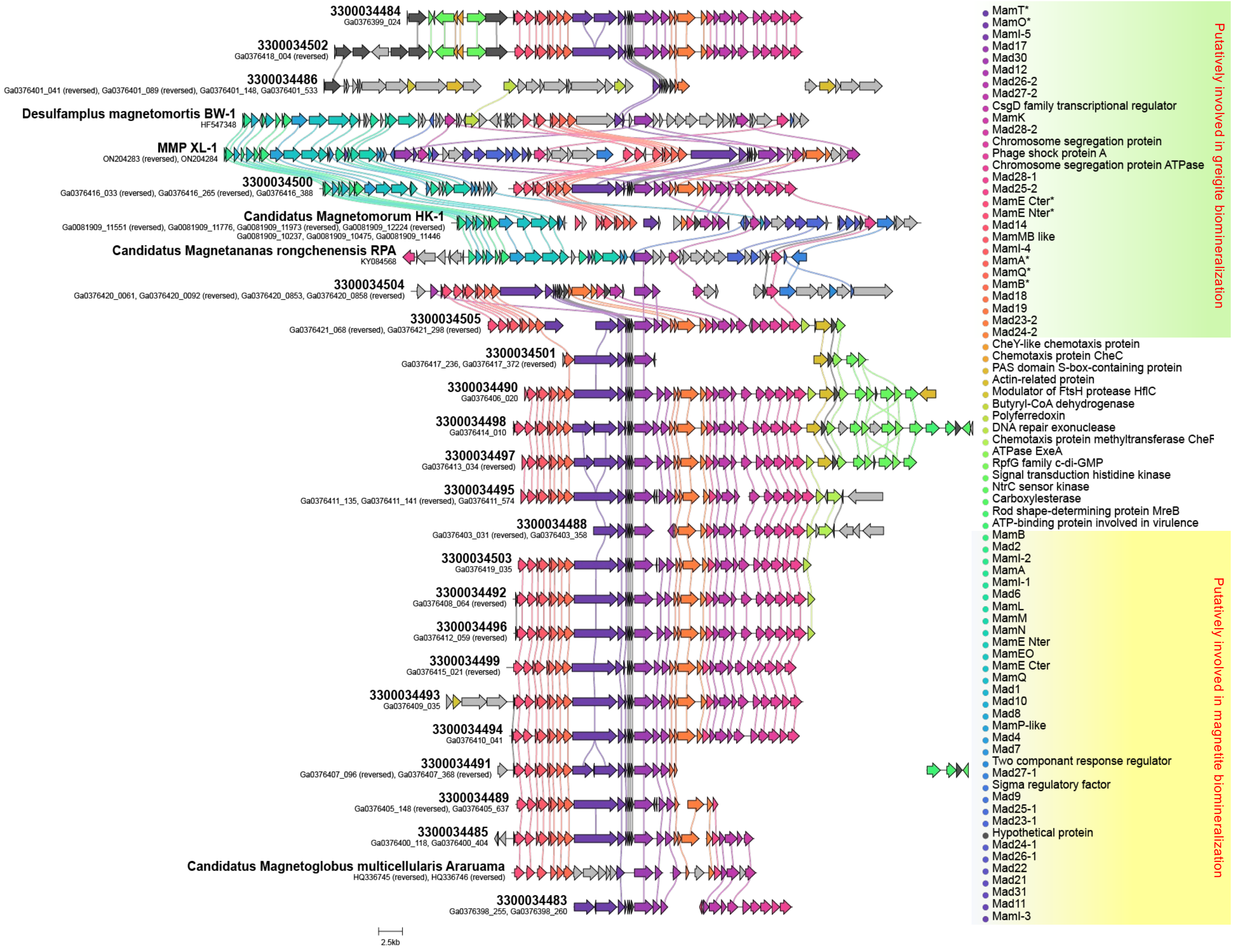

Fig. S19. Gene synteny for scaffolds containing the magnetosome gene clusters compared. The corresponding annotations of colored genes are shown in the legend to the right.
