# Supplementary material for: Multicellular magnetotactic bacteria are genetically heterogeneous consortia with metabolically differentiated cells
Source: PLoS Biol. 2024 Jul 11;22(7):e3002638. doi: 10.1371/journal.pbio.3002638 (PMC11239054; doi:10.1371/journal.pbio.3002638)
Supplement: S1 Appendix — (DOCX) [file pbio.3002638.s020.docx]

**Supporting Information for**

**Multicellular magnetotactic bacteria are genetically heterogeneous consortia with metabolically differentiated cells**

George A. Schaible ^1,2^, Zackary J. Jay ^1,2,3^, John Cliff ^4,#^, Frederik Schulz ^5^, Colin Gauvin ^2,3^, Danielle Goudeau ^5^, Rex R. Malmstrom ^5^, S. Emil Ruff ^6^, Virginia Edgcomb ^7^, and Roland Hatzenpichler ^1,2,3,8,*^

**SI Results and discussion**

**Protologue**

We assign type genomes for eight newly discovered species of MMB and propose the following provisional taxonomic assignments. All researchers were contacted and gave permission to name new MMB species after them. See Table D in S2 Appendix.

- ***Candidatus* Magnetoglobus abreuianus sp. nov.**

a.bre.u.i.a'nus N.L. masc. adj. abreuianus; named in honor of Fernanda Abreu, who described the first species of MMB, Magnetoglobus multicellularis (1). This uncultured species is represented by bin 3300034485, which has an estimated completeness of 89.22%, a contamination of 2.09%, with no 16S rRNA, 23S rRNA or 5S rRNA genes.

- ***Candidatus* Magnetoglobus debarrosii sp. nov.**

de.bar.ro'si.i N.L. gen. n. debarrosii, of de Barros; named in honor of Henrique Lins de Barros, who shaped understanding of MMB for the past four decades. This uncultured species is represented by bin 3300034500, which has an estimated completeness of 90.62%, a contamination of 1.94%, and contains 16S rRNA, 23S rRNA and 5S rRNA genes.

- ***Candidatus* Magnetoglobus farinai sp. nov.**

fa.ri.'na.i N.L. gen. n. farinai, of Farina; named in honor of Marcos Farina, who co-discovered MMB in 1983 (2, 3). This uncultured species is represented by bin 3300034494, which has an estimated completeness of 90.65%, a contamination of 0.86%, and contains 16S rRNA, 23S rRNA and 5S rRNA genes.

- ***Candidatus* Magnetoglobus keimiae sp. nov.**

ke.i'mi.ae N.L. gen. n. keimiae, of Keim; named in honor of Carolina Keim, who first demonstrated the multicellular life cycle of MMB (4). This uncultured species is represented by bin 3300034495, which has an estimated completeness of 94.77%, a contamination of 1.53%, and contains 16S rRNA, 23S rRNA and 5S rRNA genes.

- ***Candidatus* Magnetoglobus linsii sp. nov.**

lin'si.i N.L. gen. n. linsii, of Lins; named in honor of the late Ulysses Lins, whose pursuit of pure, “romantic” scientific questions (5) shaped our understanding of MMB. This uncultured species is represented by bin 3300034496, which has an estimated completeness of 93.56%, a contamination of 1.29%, and contains 16S rRNA, 23S rRNA and 5S rRNA genes.

- ***Candidatus* Magnetoglobus martinsiae sp. nov.**

mar.tin'si.ae N.L. gen. n. martinsiae, of Martins; named in honor of Juliana Lopes Martins’ contributions to the study of MMB. This uncultured species is represented by bin 330034493, which has an estimated completeness of 91.77%, a contamination of 0.86%, with no 16S rRNA, 23S rRNA or 5S rRNA genes.

- ***Candidatus* Magnetoglobus simmonsiae sp. nov.**

sim.mon'si.ae N.L. gen. n. simmonsiae, of Simmons; named in honor of Sherri Simmons, whose research on MMB in Little Sippewissett Salt Marsh laid the foundation for much of our analysis (6). This uncultured species is represented by bin 3300034505, which has an estimated completeness of 85.37%, a contamination of 1.31%, and contains 16S rRNA, 23S rRNA and 5S rRNA genes.

- ***Candidatus* Magnetomorum sippewissettense sp. nov.**

sip.pe.wis.set.ten'se N.L. neut. adj. sippewissettense; pertaining to Sippewissett, named after Little Sippewissett Salt Marsh, Falmouth, MA, USA, where this study was conducted. This uncultured species is represented by bin 3300034504, which has an estimated completeness of 86.63%, a contamination of 0.32%, and contains 16S rRNA, 23S rRNA and 5S rRNA genes.

**Failure to establish an enrichment culture**

Previous studies have attempted to cultivate magnetically enriched MMB in defined media but so far there has been no success despite the ability to magnetically enrich them to >99% purity (7, 8). In an attempt to bring MMB into cultivation, we designed a medium ( Table M in S2 Appendix) informed by the geochemical composition of the water at LSSM (Table N in S2 Appendix), the metabolic predictions derived from genomic data (Fig 4 and Table G in S2 Appendix), and the results of SIP-NanoSIMS experiments (Fig 5 and Table J in S2 Appendix). Incubations were performed under anoxic conditions at 27 ºC and a pH of 7.4. MMB were found to maintain their magnetotaxis and could be recovered from the media for up to 15 days, after which no MMB could be magnetically enriched nor identified using FISH.

**Characterization of magnetosome and light sensing genes**

Previous spectroscopic analysis has indicated the utilization of greigite magnetosomes in LSSM MMB (9), though genes relating to greigite production in LSSM MMB have not previously been identified. Genomic analysis of MMB from other locations (i.e., German Wadden Sea) revealed they are capable of synthesizing magnetite and/or greigite within their magnetosome, though greigite is most common due to environmental thermodynamic restrictions (10-13). We identified core greigite biomineralization genes in all single consortia metagenomes (SCMs) (*mamA*, B*, E-Cter*, E-Nter*, I-4*, I-5*, MB-like*, O*, Q*,* and *T** as well as *mad12, 14, 17-19, 23-30,* and *mamK)* and magnetite biomineralization genes in SCM 3300034500. The organization of the magnetosome gene clusters (MGCs) was conserved across LSSM SCMs. The synteny of the greigite biomineralizing genes were similar to *Ca*. Magnetoglobus multicellularis and MMP XL-1, although *Ca*. M. sippewissettense appears to lack the organization found in *Ca*. *Magnetoglobus* species. The synteny of magnetite biomineralizing genes in 3300034500 was conserved across *Ca*. Magnetomorum HK-1, *Ca*. Magnetananas rongchenensis RPA, MMP XL-1, and *Desulfamplus magnetomortis* BW-1 (S19 Fig). Greigite and magnetite synthesizing genes have been identified in the genomes of aforementioned MMB but greigite appears to be preferentially used over magnetite (10, 11), which is congruent with observations of LSSM MMB (S7 Fig). An explanation for the presence of magnetite biomineralizing genes in 3300034500 could be horizontal gene transfer (14), although their function/role in the environment is unclear. The SCM MGCs contained additional genes surrounding the core greigite magnetosome genes including genes encoding for actin-related proteins, rod shape-determining protein MreB, and chemotaxis protein CheF, all potentially involved in the formation and maintenance of the magnetosome (S19 Fig and Table O in S2 Appendix).

Genomic and *in vitro* observations indicate light plays an important role in the behavior and position of MMB in the sediment column and has even been shown to be responsible for triggering cell division (7, 15, 16). The *kaiB* and *kaiC* genes, involved in circadian cycle, and genes for bacteriophytochrome and photoactive yellow protein were recovered from the SCMs (Table G in S2 Appendix), supporting previous observations of LSSM MMB response to light (15). In addition, multiple copies of two-component chemotaxis genes were identified in the SCMs. The combination of genes related to magnetotaxis, phototaxis, and chemotaxis likely enables MMB to effectively navigate environmental gradients. Moreover, the identification of genes protecting against oxygen radicals (Table G in S2 Appendix) implies MMB are potentially capable of survival in (micro)oxic sediment layers. Taken together, our finding suggests LSSM MMB likely maintain constant movement along chemical gradients in their surroundings, as has been previously suggested (7).

**SI Materials and methods**

**Sample collection and magnetic enrichment of MMB**

Sediment samples were collected from a tidal pool at Little Sippewissett salt marsh (LSSM, 41.5758762, -70.6393191) in Falmouth, MA (USA) during low tide on October 2^nd^ 2018, August 17^th^ 2020, September 21^st^ 2021, and August 28^th^ 2022. For each sample, 1 L of sediment slurry (7:3 sediment to water ratio) was collected in plastic bottles and shipped within one day on ice to Montana State University, Bozeman, MT (USA), where the slurry was transferred to a 1 L glass beaker and stored in the dark at ambient laboratory temperature (~23ºC). MMB were magnetically enriched from the sediment by placing the South end of a magnetic stir bar against the exterior of the glass beaker just above the sediment layer, agitating the sediment by stirring, and then allowing the sediment to settle for 60 minutes. Magnetically enriched MMB were collected by pipette and further enriched as previously described (9) (SI Video 1).

**Scanning electron microscopy (SEM) and cellulase experiment**

To acquire SEM micrographs of MMB, a Zeiss (Jena, Germany) SUPRA 55VP field emission scanning electron microscope (FE-SEM) was operated at 1 keV under a 0.2–0.3 mPa vacuum with a working distance of 5 mm and 30 μm aperture. For the cellulase experiment, samples of magnetically enriched MMB were incubated for 1 hr at 37°C in 0.22 µm filtered LSSM water with a pH adjusted to 5 for optimal cellulase activity. MMB were treated with 5 mg/mL of cellulase (MP Biomedicals, Solon, OH USA) as per the manufacturer’s instructions. A control reaction under the same conditions but without cellulase was performed to check the effect of temperature and low pH on MMB. The incubation was stopped by the addition of PFA to a final concentration of 4% and samples incubated at ambient temperature for 1 hr, after which cells were centrifuged at 16,000 g for 5 minutes and the supernatant removed, and cells resuspended in 1x PBS. Cells were dried onto a mirrored stainless-steel slide and dried at 46 °C for 2 minutes, after which they were washed in MilliQ water three times for 10 seconds each and the slide was air dried. All electron microscopy work was performed at the Imagining and Chemical Analysis Laboratory (ICAL) of Montana State University (Bozeman, MT). No conductivity coating was applied prior to analysis.

**Phylogenetic, Phylogenomic, and Comparative Genomic analyses**

The 16S rRNA gene sequences encoded in the MMB SCMs were used in BLASTn (17) searches to screen the NCBI database for related sequences (Table B in S2 Appendix). All 16S rRNA sequences were aligned using SSU-ALIGN and a maximum likelihood analysis was performed using FastTree2.1 with 500 ultrafast bootstraps (18, 19). Of 139 single-copy bacterial genes searched (20), a subset of six were present in all 22 SCM (Table C in S2 Appendix). These were aligned with reference sequences using MUSCLE (21), concatenated, and phylogenetically analyzed with FastTree2.1 (500 ultrafast bootstraps) (18). Average nucleotide identities (ANIs) of SCMs and 16S rRNA sequences were calculated with FastANI (22) and pairwise BLASTn comparisons, respectively.

**Genome annotation**

The metabolic potential of MMB SCMs was determined by mapping gene annotations provided by IMG/M (23) to metabolic pathways outlined in the KEGG (Kyoto Encyclopedia of Genes and Genomes) database (24). Further investigation of genes was done by inspection of gene neighborhoods and identification of conserved domains and motifs through submission of genes to the NCBI conserved domain database (25) and MPI Bioinformatics HHpred Toolkit (26). Classification of hydrogenases was done using HydDB (27) and if a subunit is membrane bound or soluble determined using DeepTMHMM (28).

**Comparative genomic analysis of magnetosome gene clusters**

To identify the magnetosome gene clusters, pairwise BLASTn comparisons of individual magnetosome genes from *Ca*. Desulfamplus magnetomortis BW-1 (HF547348) (29) were performed on each of the individual SCMs as well as the reference genomes of *Ca*. Magnetoglobus multicellularis (IMG ID 2558860350) (7) and *Ca*. Magnetomorum sp. HK-1 (IMG ID 2648501189) (11). Gene synteny figures of magnetosome encoding loci were made with Clinker (v0.0.27) using default settings and an identity setting of 0.45 (30).

**Fluorescence *in situ* hybridization (FISH)**

Double-labeled oligonucleotide probes for FISH (DOPE-FISH, (31)) were purchased from Integrated DNA Technologies (Coralville, IA) to visualize different MMB taxa. Genus level populations of MMB were targeted by using newly designed DOPE-FISH probes targeting the 1032-1049 nt region of the 16S rRNA (*E. coli* equivalent) using full length 16S rRNA gene sequences from the MMB SCMs and previously published 16S RNA gene clone sequences from LSSM (6) and the two reference genomes. Probes were designed to target five genus level populations of MMB in LSSM (groups 1-5) as well as three individual species within groups 1 and 2 (S9 Fig and Table I in S2 Appendix). Probes were designed manually using ARB (32) and evaluated *in silico* using the TestProbe tool of Silva ((33), <http://arb-silva.de>, database release 138.1), the MatchProbe tool of ARB, and mathFISH ((34), <http://mathfish.cee.wisc.edu/>). All probes have at least one central mismatch to non-target sequences (Table I in S2 Appendix) and were verified in the Silva database (33). To ensure stringency of each probe, competitor probes were designed for each probe and used accordingly. Group-specific probes were designed to compete for the same binding site to guarantee specific binding. Specificity of genus-specific probes was checked using hybridization curve assays in CloneFISH (35) experiments using representative sequences for each of the five MMB groups. Fixed cells were dehydrated using an increasing ethanol series (1 min in each 50, 80, and 96% ethanol) and FISH was carried out on Teflon coated glass slides. Samples were hybridized for three hours in a humid chamber at 46 °C with a final probe concentration of 2.5 ng μL^−1^. Positive and negative controls using EUB338 and NonEUB338 (36) were conducted routinely. Neither in CloneFISH nor in environmental FISH experiments, *E. coli* cells or MMB, respectively, were labeled by more than one MMB group- or species-specific probe, demonstrating specificity of the newly designed probes at the final formamide concentrations (Table I in S2 Appendix).

**Bioorthogonal noncanonical amino acid tagging (BONCAT) and confocal fluorescence microscopy**

To evaluate the activity of MMB within LSSM, BONCAT incubations were performed on LSSM sediments. A 15 cm long sediment core was collected on August 17^th^ 2020 from the West end of the sample site and shipped to MSU overnight. Upon receipt, the core was sectioned into 1 cm horizons that were homogenized and divided into triplicate 25 mL serum vials. Vials were placed in an anoxic chamber (Coy Lab Products, Grass Lake, MI) and 10 mL of 0.22 µm filtered LSSM water (made anoxic by bubbling with nitrogen gas for 60 minutes) added to each vial. Samples were amended with 50 μM L-Homopropargylglycine (HPG, Click Chemistry Tools, Scottsdale, AZ) except for triplicate negative controls. Samples were incubated for 24 hours in the dark at ambient lab temperature, after which MMB were magnetically enriched from each triplicate horizon incubation and fixed in 4% PFA. Cells were centrifuged for 5 minutes at 16,000 g, after which the supernatant was removed, and the cell pellets resuspended in 50 µL 1× PBS and stored at 4 °C. To fluorescently label alkyne-tagged proteins, cells were dried to a glass slide and dehydrated using an ethanol series (50, 80, and 96% for three minutes each). Click chemistry using AlexaFlour-405-Azide was performed according to published methods (37). In addition, DOPE-FISH was performed on the samples to identify individual Groups of MMB (see SI). Cells were imaged using a Leica DM4B epifluorescent microscope (Leica Microsystems, Deerfield, IL USA) and relative fluorescence intensity calculated using Daime with normal edge thresholding settings (38).

To evaluate differences in activity within individual MMB consortia, sediments containing MMB were amended with 50 µM *L*-azidohomoalanine (AHA, Click Chemistry Tools, Scottsdale, AZ USA) and incubated at ambient temperature in the dark for 24 hours, after which the MMB were magnetically enriched and fixed in 4% PFA for 60 minutes at ambient temperature. Cells were centrifuged for 5 minutes at 16,000 g, after which the supernatant was removed, and the cell pellets resuspended in 50 µL 1× PBS and stored at 4 °C. To fluorescently tag azide-labeled proteins, cells were dried to a glass slide and dehydrated using an ethanol series (50, 80, and 96% for three minutes each). Click chemistry using AlexaFlour-488-Alkyne was performed using published methods (37).

**Confocal Raman microspectroscopy and spectral processing**

Raman spectra of individual MMB were acquired using a LabRAM HR Evolution Confocal Raman microscope (Horiba Jobin-Yvon) equipped with a 532 nm laser and 300 grooves/mm diffraction grating. Spectra of the MMB were acquired using a 100× dry objective (NA = 0.9), with 10 acquisitions of 2 seconds each, and a laser power of 4.5 mW. Spectra were processed using LabSpec version 6.5.1.24 (Horiba) with a Savitsky-Goly smoothing algorithm, baselined, and finally normalized to the maximum intensity within the 2,800-3,100 cm^-1^ regions. Peaks corresponding to lipids, PHB, and exopolysaccharides were identified in previous studies (39, 40) and are listed in Table H in S2 Appendix.

**NanoSIMS**

Ion images were acquired using the NanoSIMS 50L (Cameca) at the Environmental Molecular Sciences Laboratory at the Pacific Northwest National Laboratory. All NanoSIMS images were acquired using a 16 keV Cs+ primary ion beam at 512 × 512-pixel resolution with a dwell time of 13.5 ms px^−1^. Analysis areas were pre-sputtered with ~ 1016 ions cm^−2^ prior to analysis. Secondary ions were accelerated to 8 keV and counted simultaneously using electron multipliers (EMs). The vacuum gauge pressure in the analytical chamber during all analyses was consistently less than 3 × 10^−10^ mbar. Other analytical conditions included a 200 μm D1 aperture, 30 μm entrance slit, 350 μm aperture slit, and 100 μm exit slits. The OpenMIMS plugin for ImageJ was used to access and correct images pixel by pixel for dead time (44 ns) and QSA (β = 0.5). HSI images shown in main text are filtered with a median filter ratio radius of 0.5. This filter is used to improve contrast but does not adversely affect quantitative data reported in tabular form for the regions of interest (ROIs). Data from regions of interest (ROIs) were exported to a custom spreadsheet for data reduction. Quantitative ^13^C^12^C/^12^C_2_ analyses were calibrated against an in-house yeast reference material of known natural abundance δ^13^C during the same analytical session using similar conditions to those used to analyze the bacterial culture samples. An unknown background signal interfering with the ^2^HC signal was subtracted using the yeast ion images but no attempt was made to calibrate the ^2^HC/^1^HC. These data are therefore not strictly quantitative, but this does not change interpretation of the relatively higher ^2^H content of the enriched samples compared with controls (Schaible, Cliff, *et al.*, manuscript in preparation). The yeast reference material had been stored in the NanoSIMS under high vacuum for several months prior to the analyses reported here. During ^2^HC/^1^HC analyses, detectors collecting secondary ^2^HC and ^1^HC ions were situated near the center of the magnet radius and Helmholtz steering coils were carefully adjusted to improve simultaneous secondary centering characteristics. Propagation of uncertainty includes counting statistics and external precision of isotopic ratios of 16 individual yeast cells.

**Geochemical analysis**

Overlaying water from LSSM was collected and 0.22 μm filtered into 50 mL tubes for ion chromatography and inductively coupled plasma optical emission spectroscopy (ICP-OES). Trace-metal grade HNO_3_ was added to the ICP-OES tubes for a final concentration of 2%. Samples for total organic carbon (TOC) were collected by 0.22 μm filtering LSSM water into ashed glass vials. All geochemical measurements were made in the Environmental Analytical Laboratory at Montana State University (Bozeman, Montana). Details on how chemical analyses were performed can be found in Lynes, Krukenberg et al 2023 (41).

**Statistical analysis**

All datasets were analyzed in R (42) using the tidyverse, rstatix, and ggpubr packages (43, 44). Statistical differences between multiple variables were determined using ANOVA and pairwise t-tests with a Bonferroni p-adjusted method. Boxplots show the distribution of the dataset, where the box corresponds to the interquartile range (IQR) containing the middle 50% of the data, the black line inside the box represents the median, and the whiskers extend to the minimum and maximum values within 1.5 times the IQR from the first and third quartiles, respectively.

**References**

1. Abreu F, Martins JL, Silveira TS, Keim CN, de Barros HG, Filho FJ, et al. 'Candidatus Magnetoglobus multicellularis', a multicellular, magnetotactic prokaryote from a hypersaline environment. Int J Syst Evol Microbiol. 2007;57(Pt 6):1318-22.

2. Esquivel DMS, Lins de Barros HGP, Farina M, Aragão PHA, Danon J. Microorganismes magnétotactiques de la region de Rio de Janeiro. Biology of the cell. 1983;47:227-34.

3. Farina M, Lins de Barros H, Esquivel DMS, Danon J. Ultrastructure of a magnetotactic bacterium. Biol Cell. 1983;48:85-8.

4. Keim CN, Martins JL, Abreu F, Rosado AS, de Barros HL, Borojevic R, et al. Multicellular life cycle of magnetotactic prokaryotes. FEMS Microbiol Letters. 2004;240(2):203-8.

5. Keim CN, Farina M, Lins U. Magnetoglobus, Magnetic Aggregates in Anaerobic Environments. Microbe 2. 2007:437-45.

6. Simmons SL, Edwards KJ. Unexpected diversity in populations of the many-celled magnetotactic prokaryote. Environ Microbiol. 2007;9(1):206-15.

7. Abreu F, Morillo V, Nascimento FF, Werneck C, Cantao ME, Ciapina LP, et al. Deciphering unusual uncultured magnetotactic multicellular prokaryotes through genomics. ISME J. 2014;8(5):1055-68.

8. Wenter R, Wanner G, Schuler D, Overmann J. Ultrastructure, tactic behaviour and potential for sulfate reduction of a novel multicellular magnetotactic prokaryote from North Sea sediments. Environ Microbiol. 2009;11(6):1493-505.

9. Schaible GA, Kohtz AJ, Cliff J, Hatzenpichler R. Correlative SIP-FISH-Raman-SEM-NanoSIMS links identity, morphology, biochemistry, and physiology of environmental microbes. ISME Communications. 2022;2(1).

10. Cui K, Pan H, Chen J, Liu J, Zhao Y, Chen S, et al. A Novel Isolate of Spherical Multicellular Magnetotactic Prokaryotes Has Two Magnetosome Gene Clusters and Synthesizes Both Magnetite and Greigite Crystals. Microorganisms. 2022;10(5).

11. Kolinko S, Richter M, Glockner FO, Brachmann A, Schuler D. Single-cell genomics reveals potential for magnetite and greigite biomineralization in an uncultivated multicellular magnetotactic prokaryote. Environ Microbiol Rep. 2014;6(5):524-31.

12. Leao P, Chen YR, Abreu F, Wang M, Zhang WJ, Zhou K, et al. Ultrastructure of ellipsoidal magnetotactic multicellular prokaryotes depicts their complex assemblage and cellular polarity in the context of magnetotaxis. Environ Microbiol. 2017;19(6):2151-63.

13. Simmons SL, Edwards KJ. Geobiology of magnetotactic bacteria. Magnetoreception and magnetosomes in bacteria: Springer; 2006. p. 77-102.

14. Lefevre CT, Trubitsyn D, Abreu F, Kolinko S, de Almeida LG, de Vasconcelos AT, et al. Monophyletic origin of magnetotaxis and the first magnetosomes. Environ Microbiol. 2013;15(8):2267-74.

15. Shapiro OH, Hatzenpichler R, Buckley DH, Zinder SH, Orphan VJ. Multicellular photo-magnetotactic bacteria. Env Microbiol Rep. 2011;3(2):233-8.

16. Qian X, Zhao Y, Santini C-L, Pan H, Xiao T, Chen H, et al. How light affect the magnetotactic behavior and reproduction of ellipsoidal multicellular magnetoglobules? Journal of Oceanology and Limnology. 2021;39(6):2005-14.

17. Altschul SF, Gish W, Miller W, Myers EW, Lipman DJ. Basic local alignment search tool. Journal of molecular biology. 1990;215(3):403-10.

18. Price MN, Dehal PS, Arkin AP. FastTree 2–approximately maximum-likelihood trees for large alignments. PloS one. 2010;5(3):e9490.

19. Nawrocki EP, Kolbe DL, Eddy SR. Infernal 1.0: inference of RNA alignments. Bioinformatics. 2009;25(10):1335-7.

20. Campbell JH, O'Donoghue P, Campbell AG, Schwientek P, Sczyrba A, Woyke T, et al. UGA is an additional glycine codon in uncultured SR1 bacteria from the human microbiota. Proc Natl Acad Sci U S A. 2013;110(14):5540-5.

21. Edgar RC. MUSCLE: multiple sequence alignment with high accuracy and high throughput. Nucleic Acids Res. 2004;32(5):1792-7.

22. Jain C, Rodriguez RL, Phillippy AM, Konstantinidis KT, Aluru S. High throughput ANI analysis of 90K prokaryotic genomes reveals clear species boundaries. Nat Commun. 2018;9(1):5114.

23. Chen IA, Chu K, Palaniappan K, Ratner A, Huang J, Huntemann M, et al. The IMG/M data management and analysis system v. 7: content updates and new features. Nucleic Acids Research. 2023;51(D1):D723-D32.

24. Kanehisa M, Furumichi M, Sato Y, Kawashima M, Ishiguro-Watanabe M. KEGG for taxonomy-based analysis of pathways and genomes. Nucleic Acids Res. 2023;51(D1):D587-D92.

25. Lu S, Wang J, Chitsaz F, Derbyshire MK, Geer RC, Gonzales NR, et al. CDD/SPARCLE: the conserved domain database in 2020. Nucleic Acids Res. 2020;48(D1):D265-D8.

26. Zimmermann L, Stephens A, Nam SZ, Rau D, Kubler J, Lozajic M, et al. A Completely Reimplemented MPI Bioinformatics Toolkit with a New HHpred Server at its Core. J Mol Biol. 2018;430(15):2237-43.

27. Sondergaard D, Pedersen CN, Greening C. HydDB: A web tool for hydrogenase classification and analysis. Sci Rep. 2016;6:34212.

28. Hallgren J, Tsirigos KD, Pedersen MD, Almagro Armenteros JJ, Marcatili P, Nielsen H, et al. DeepTMHMM predicts alpha and beta transmembrane proteins using deep neural networks bioRxiv. 2022.

29. Lefevre CT, Trubitsyn D, Abreu F, Kolinko S, Jogler C, de Almeida LG, et al. Comparative genomic analysis of magnetotactic bacteria from the Deltaproteobacteria provides new insights into magnetite and greigite magnetosome genes required for magnetotaxis. Environ Microbiol. 2013;15(10):2712-35.

30. Gilchrist CLM, Chooi YH. clinker & clustermap.js: automatic generation of gene cluster comparison figures. Bioinformatics. 2021;37(16):2473-5.

31. Stoecker K, Dorninger C, Daims H, Wagner M. Double labeling of oligonucleotide probes for fluorescence in situ hybridization (DOPE-FISH) improves signal intensity and increases rRNA accessibility. Appl Environ Microbiol. 2010;76(3):922-6.

32. Ludwig W, Strunk O, Westram R, Richter L, Meier H, Yadhukumar, et al. ARB: a software environment for sequence data. Nucleic Acids Res. 2004;32(4):1363-71.

33. Quast C, Pruesse E, Yilmaz P, Gerken J, Schweer T, Yarza P, et al. The SILVA ribosomal RNA gene database project: improved data processing and web-based tools. Nucleic Acids Res. 2013;41(Database issue):D590-6.

34. Yilmaz LS, Parnerkar S, Noguera DR. mathFISH, a web tool that uses thermodynamics-based mathematical models for in silico evaluation of oligonucleotide probes for fluorescence in situ hybridization. Appl Environ Microbiol. 2011;77(3):1118-22.

35. Schramm A, Fuchs, B. M., Nielsen, J. L., Tonolla, M., & Stahl, D. A. Fluorescence in situ hybridization of 16S rRNA gene clones (Clone‐FISH) for probe validation and screening of clone libraries. Environ Microbiol. 2002;4:713-20.

36. Daims H, Brühl A, Amann R, Schleifer K-H, Wagner M. The Domain-specific Probe EUB338 is Insufficient for the Detection of all Bacteria: Development and Evaluation of a more Comprehensive Probe Set. Systematic and Applied Microbiology. 1999;22(3):434-44.

37. Hatzenpichler R, Scheller S, Tavormina PL, Babin BM, Tirrell DA, Orphan VJ. In situ visualization of newly synthesized proteins in environmental microbes using amino acid tagging and click chemistry. Environ Microbiol. 2014;16(8):2568-90.

38. Daims H, Lucker S, Wagner M. daime, a novel image analysis program for microbial ecology and biofilm research. Environ Microbiol. 2006;8(2):200-13.

39. Wang Y, Huang WE, Cui L, Wagner M. Single cell stable isotope probing in microbiology using Raman microspectroscopy. Curr Opin Biotechnol. 2016;41:34-42.

40. Brezeștean I, Bocăneală M, Gherman AMR, Porav SA, Kacsó I, Rakosy-Tican E, et al. Spectroscopic investigation of exopolysaccharides purified from Arthrospira platensis cultures as potential bioresources. Journal of Molecular Structure. 2021;1246.

41. Lynes MM, Krukenberg V, Jay ZJ, Kohtz AJ, Gobrogge CA, Spietz RL, et al. Diversity and function of methyl-coenzyme M reductase-encoding archaea in Yellowstone hot springs revealed by metagenomics and mesocosm experiments. ISME Commun. 2023;3(1):22.

42. Team RC. A langauge and environment for statistical computing Vienna, Austria: R Foundation for Statistical Computing: <http://R-project.org/>; 2023 [

43. McNamara A. Key attributes of a modern statistical computing tool. The American Statistician. 2018.

44. Kassambara A. Comparing groups: Numerical variables: Datanovia; 2019.
