# Supplementary material for: Multicellular magnetotactic bacteria are genetically heterogeneous consortia with metabolically differentiated cells
Source: PLoS Biol. 2024 Jul 11;22(7):e3002638. doi: 10.1371/journal.pbio.3002638 (PMC11239054; doi:10.1371/journal.pbio.3002638)

Fig. S2. Phylogenetic analysis of MMB using near-full length 16S rRNA genes (length listed next to name) found in 14 of the 22 SCMs and in reference genomes. Tree reconstructed using maximum likelihood method with bootstrap values calculated using 500 replicates. Bootstrap values above 50 are shown. *Ca.* M. abreuianus is not shown in this analysis because no 16S rRNA gene was recovered from the SCM. Color coded sequences belong to their respective SCM, as shown in Table A in S2 Appendix. Bars on right show specificities of our newly designed FISH probes that target genus-level groups of MMB in LSSM (Table I in S2 Appendix).

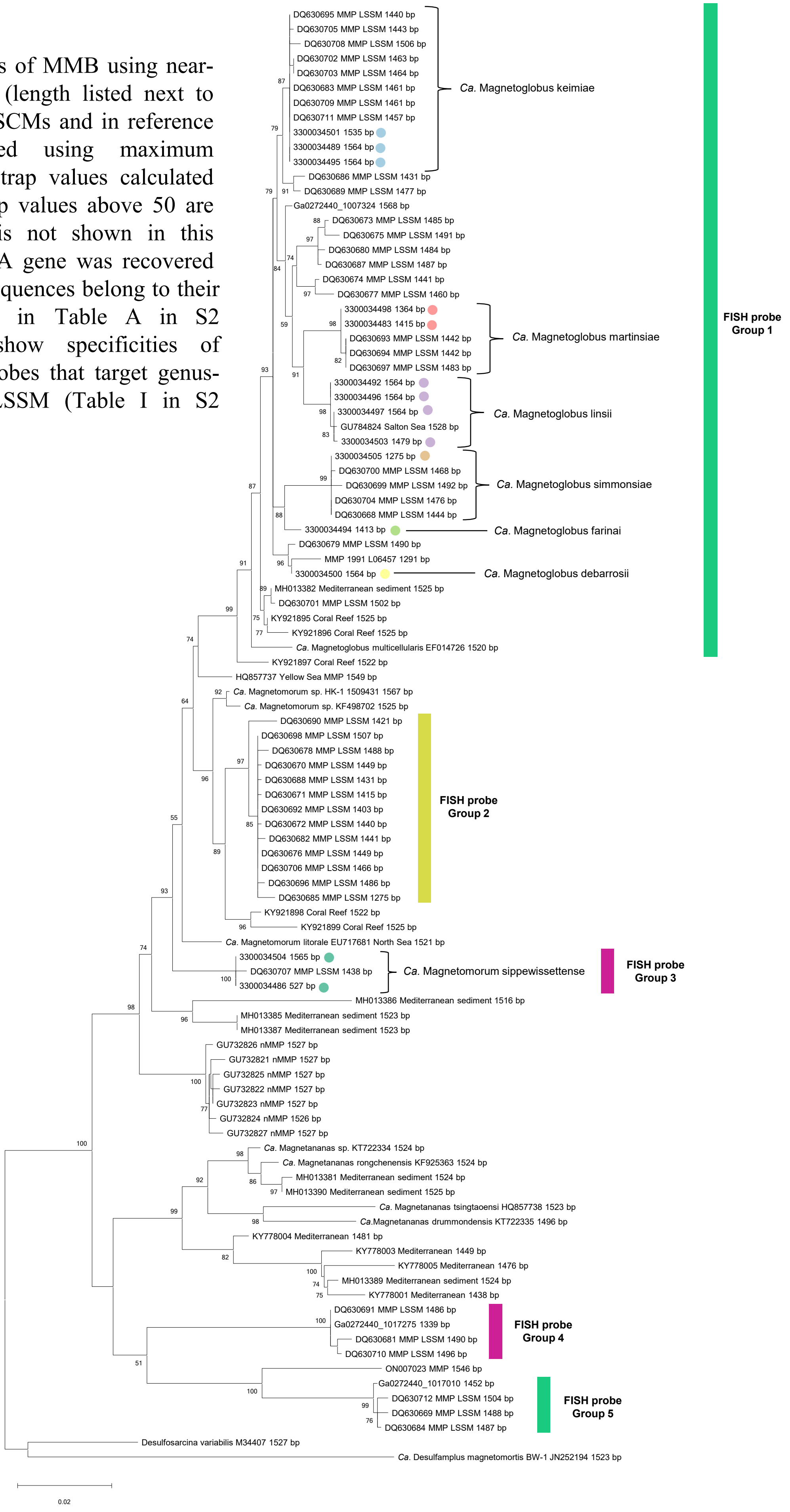

Supplement: S2 Fig — Tree reconstructed using maximum likelihood method with bootstrap values calculated using 500 replicates. Bootstrap values above 50 are shown. Ca. M. abreuianus is not shown in this analysis because no 16S rRNA gene was recovered from the SCM. Color coded sequences belong to their respective SCM, as shown in Table A in S2 Appendix. Bars on right show specificities of our newly designed FISH probes that target genus-level groups of MMB in LSSM (Table I in S2 Appendix). (PDF) [file pbio.3002638.s002.pdf]
