# Supplementary material for: Multicellular magnetotactic bacteria are genetically heterogeneous consortia with metabolically differentiated cells
Source: PLoS Biol. 2024 Jul 11;22(7):e3002638. doi: 10.1371/journal.pbio.3002638 (PMC11239054; doi:10.1371/journal.pbio.3002638)
Supplement: S3 Fig — Percent identity values are shown within boxes. Bars on left highlight MMB groups for which genus-level FISH probes designed (Table I in S2 Appendix). (PDF) [file pbio.3002638.s003.pdf]

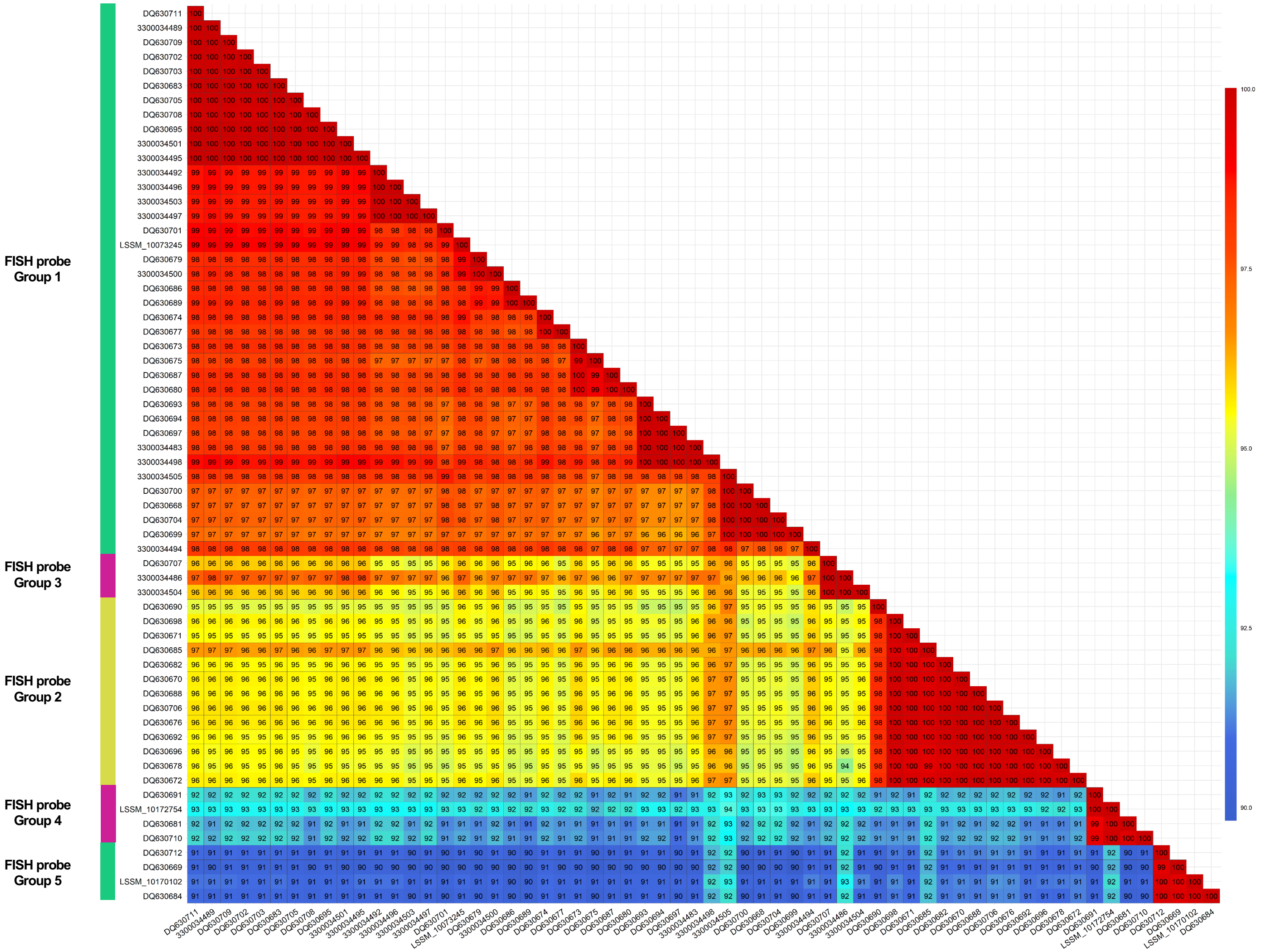

Fig. S3. Near-full length 16S rRNA gene comparison of all sequences recovered in this study and previous studies at LSSM (Simmons and Edwards 2007). Percent identity values are shown within boxes. Bars on left highlight MMB groups for which genus-level FISH probes designed (Table I in S2 Appendix).
